# Supplementary material for: Increased photosynthesis during spring drought in energy-limited ecosystems
Source: Nat Commun. 2023 Nov 29;14:7828. doi: 10.1038/s41467-023-43430-9 (PMC10687245; doi:10.1038/s41467-023-43430-9)
Supplement: Supplementary file 1 — Supplementary Information [file 41467_2023_43430_MOESM1_ESM.pdf]

Increased photosynthesis during spring drought in energy-limited ecosystems

David L. Miller[1], Sebastian Wolf[2], Joshua B. Fisher[3], Benjamin F. Zaitchik[4], Jingfeng Xiao[5], and Trevor F. Keenan[1,6]

1. Department of Environmental Science, Policy, and Management, University of California, Berkeley, California 94720, USA
2. Department of Environmental Systems Science, ETH Zurich, 8092 Zurich, Switzerland
3. Schmid College of Science and Technology, Chapman University, 1 University Drive, Orange, California, 92866, USA
4. Department of Earth and Planetary Sciences, The Johns Hopkins University, Baltimore, Maryland 21218, USA
5. Earth Systems Research Center, Institute for the Study of Earth, Oceans, and Space, University of New Hampshire, Durham, NH 03824, USA
6. Climate and Ecosystem Sciences Division, Lawrence Berkeley National Laboratory, Berkeley, California 94720, USA

Supplemental Material – Figures and Tables

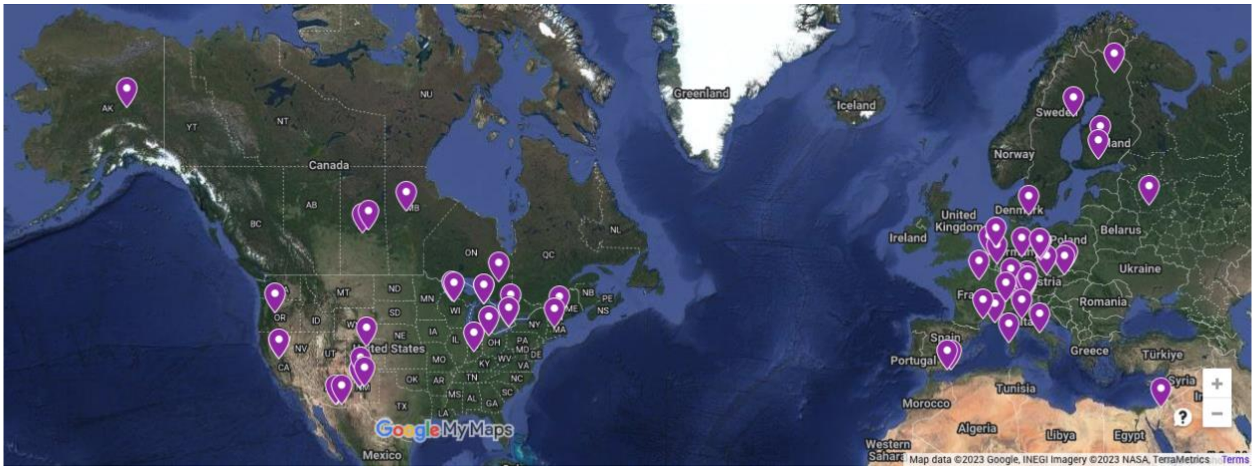

Supplemental Figure S1: Map of eddy covariance towers used for spring 10+ years analysis. Base map from Google My Maps (Map data ©2023 Google, INEGI Imagery ©2023 NASA, TerraMetrics). Also including interactive map link to Google My Maps showing spatial distribution of all eddy covariance sites: https://www.google.com/maps/d/u/0/edit?mid=1k2zEqE7gsJWOfuN8GVqZb3yYi7CrZGxi&usp=sharing

Supplemental Table S1: List of all eddy covariance sites used in analysis. Table is separated into (a) all sites with 10+ years of data in spring, (b) additional sites with 10+ years of data in summer, and (c) additional sites with 5+ years of data in all seasons. Ref column includes link to relevant reference cited at the end of the supplemental materials.

(a) Eddy covariance sites with 10+ years of data in spring (n = 61).

Site ID	Site Name	IGBP	Clim	Latitude	Longitude	Elev	WI	Data Source	Ref
**AT-Neu**	Neustift	GRA	Dfc	47.11667	11.3175	970	1.72	FLUXNET2015	1
**BE-Bra**	Brasschaat	MF	Cfb	51.30761	4.51984	16	1.20	ICOS-2020	2
**BE-Vie**	Vielsalm	MF	Cfb	50.30493	5.99812	493	1.82	ICOS-2020	3
**CA-Cbo**	Ontario - Mixed Deciduous, Borden Forest Site	DBF	Dfb	44.3167	-79.9333	120	1.28	OneFlux	4
**CA-Gro**	Ontario - Groundhog River, Boreal Mixedwood Forest	MF	Dfb	48.2167	-82.1556	340	1.35	FLUXNET2015	5
**CA-Man**	Manitoba - Northern Old Black Spruce (former BOREAS Northern Study Area)	ENF	Dfc	55.87962	-98.48081	259	1.06	FLUXNET2015	6
**CA-Oas**	Saskatchewan - Western Boreal, Mature Aspen	DBF	Dfc	53.62889	-106.19779	530	0.79	FLUXNET2015	6
**CA-Obs**	Saskatchewan - Western Boreal, Mature Black Spruce	ENF	Dfc	53.98717	-105.11779	628.94	0.86	FLUXNET2015	6
**CA-TP3**	Ontario - Turkey Point 1974 Plantation White Pine	ENF	Dfb	42.70681111	-80.34831389	184	1.30	FLUXNET2015	7
**CA-TP4**	Ontario - Turkey Point 1939 Plantation White Pine	ENF	Dfb	42.710161	-80.357376	184	1.33	OneFlux	7
**CH-Cha**	Chamau	GRA	Cfb	47.21022	8.41044	393	1.78	ICOS-2020	8
**CH-Dav**	Davos	ENF	Dfc	46.81533	9.85591	1639	2.17	ICOS-2020	9
**CH-Fru**	Fruebuel	GRA	Cfb	47.11583	8.53778	982	2.12	ICOS-2020	10
**CH-Lae**	Laegern	MF	Cfb	47.47833	8.36439	689	1.72	ICOS-2020	11

**CZ-BK1**	Bily Kriz forest	ENF	Dfb	49.50208	18.53688	875	1.80	ICOS-2020	12
**CZ-Stn**	Stitna	DBF	Cfb	49.035975	17.9699	562	1.17	ICOS-2020	13
**CZ-wet**	Trebon (CZECHWET)	WET	Dfb	49.02465	14.77035	426	1.00	ICOS-2020	14
**DE-Gri**	Grillenburg	GRA	Cfb	50.95004	13.51259	385	1.24	ICOS-2020	15
**DE-Hai**	Hainich	DBF	Cfb	51.07921	10.45217	430	1.38	ICOS-2020	16
**DE-Obe**	Oberbarenburg	ENF	Cfb	50.78666	13.72129	734	1.51	ICOS-2020	17
**DE-Tha**	Tharandt	ENF	Cfb	50.96256	13.56515	385	1.13	ICOS-2020	17
**DK-Sor**	Soroe	DBF	Cfb	55.48587	11.64464	40	1.00	ICOS-2020	18
**ES-Agu**	Aguamarga	OSH	Bsh	36.940046	-2.033208	202	0.19	ICOS-2020	19
**ES-LJu**	Llano de los Juanes	OSH	Csb	36.92659	-2.75212	1600	0.44	ICOS-2020	20
**FI-Hyy**	Hyytiala	ENF	Dfc	61.84741	24.29477	181	1.51	ICOS-2020	21
**FI-Let**	Lettosuo	ENF	Dfb	60.64183	23.95952	125	1.42	ICOS-2020	22
**FI-Sod**	Sodankyla	ENF	Dfc	67.36239	26.63859	180	1.52	FLUXNET2015	23
**FR-FBn**	Font-Blanche	MF	Csa	43.24079	5.67865	436	0.52	ICOS-2020	24
**FR-Fon**	Fontainebleau-Barbeau	DBF	Cfb	48.47636	2.7801	103	0.80	ICOS-2020	25
**FR-Pue**	Puechabon	EBF	Csa	43.7413	3.5957	270	0.55	FLUXNET2015	26
**IL-Yat**	Yatir	ENF	Csa	31.34504459	35.05198851	657	0.17	ICOS-2020	27
**IT-Col**	Collelongo	DBF	Cfb	41.84936	13.58814	1560	0.99	FLUXNET2015	28
**IT-Lav**	Lavarone	ENF	Dfb	45.9562	11.28132	1353	0.86	ICOS-2020	29
**IT-MBo**	Monte Bondone	GRA	Dfb	46.01468	11.04583	1550	0.64	ICOS-2020	30
**IT-Noe**	Arca di Noe - Le Prigionette	CSH	Csa	40.60618	8.15169	25	0.66	FLUXNET2015	31
**IT-Ren**	Renon	ENF	Dfc	46.58686	11.43369	1730	1.32	ICOS-2020	32
**IT-SRo**	San Rossore	ENF	Csa	43.72786	10.28444	6	0.83	FLUXNET2015	33
**IT-Tor**	Torgnon	GRA	Dfc	45.84444	7.57806	2160	2.80	ICOS-2020	34
**NL-Loo**	Loobos	ENF	Cfb	52.16658	5.74356	25	1.34	ICOS-2018	35
**RU-Fyo**	Fyodorovskoye	ENF	Dfb	56.46153	32.92208	265	1.37	ICOS-2020	36
**SE-Deg**	Degero	WET	Dfc	64.182029	19.556539	270	1.56	ICOS-2020	37
**US-Bar**	Bartlett Experimental Forest	DBF	Dfb	44.0646	-71.2881	272	1.92	OneFlux	38
**US-Ha1**	Harvard Forest EMS Tower (HFR1)	DBF	Dfb	42.5378	-72.1715	340	1.61	FLUXNET2015	39
**US-Me2**	Metolius mature ponderosa pine	ENF	Csb	44.4523	-121.5574	1253	0.88	OneFlux	40
**US-MMS**	Morgan Monroe State Forest	DBF	Cfa	39.3232	-86.4131	275	1.23	OneFlux	41
**US-NR1**	Niwot Ridge Forest (LTER NWT1)	ENF	Dfc	40.0329	-105.5464	3050	0.80	FLUXNET2015	42
**US-Oho**	Oak Openings	DBF	Dfa	41.5545	-83.8438	230	1.02	FLUXNET2015	43
**US-PFa**	Park Falls/WLEF	MF	Dfb	45.9459	-90.2723	470	1.29	FLUXNET2015	44
**US-Seg**	Sevilleta grassland	GRA	Bsk	34.3623	-106.702	1622	0.16	OneFlux	45
**US-Ses**	Sevilleta shrubland	OSH	Bsk	34.3349	-106.7442	1604	0.16	OneFlux	45
**US-SRG**	Santa Rita Grassland	GRA	Bsk	31.789379	-110.827675	1291	0.33	OneFlux	46
**US-SRM**	Santa Rita Mesquite	WSA	Bsk	31.8214	-110.8661	1120	0.26	FLUXNET2015	46
**US-Ton**	Tonzi Ranch	WSA	Csa	38.43093184	-120.9659907	177	0.45	FLUXNET2015	47
**US-Uaf**	University of Alaska, Fairbanks	ENF	Dwc	64.8663	-147.8555	155	0.68	OneFlux	48

**US-UMB**	Univ. of Mich. Biological Station	DBF	Dfb	45.5598	-84.7138	234	1.14	OneFlux	49
**US-UMd**	UMBS Disturbance	DBF	Dfb	45.5625	-84.6975	239	1.12	OneFlux	49
**US-Var**	Vaira Ranch- Ione	GRA	Csa	38.41329154	-120.9507564	129	0.46	FLUXNET2015	47
**US-Vcp**	Valles Caldera Ponderosa Pine	ENF	Dfb	35.8624	-106.5974	2542	0.63	OneFlux	45
**US-WCr**	Willow Creek	DBF	Dfb	45.8059	-90.0799	520	1.29	FLUXNET2015	50
**US-Wjs**	Willard Juniper Savannah	SAV	Bsk	34.4255	-105.8615	1931	0.23	OneFlux	45
**US-Wkg**	Walnut Gulch Kendall Grasslands	GRA	Bsk	31.7365	-109.9419	1531	0.23	FLUXNET2015	46

(b) Summer (n = 62) eddy covariance sites with 10+ years of data includes all sites listed in spring table (a) except CA-TP3 and ES-Agu, in addition to 3 more sites: IT-Ro2, US-GLE, and US-Mpj. Fall (n = 63) eddy covariance sites with 10+ years of data includes all sites listed in spring (a) and summer (b) tables except CA-TP3.

Site ID	Site Name	IGBP	Clim	Latitude	Longitude	Elev	WI	Data Source	Ref
**IT-Ro2**	Roccarespampani 2	DBF	Csa	42.39026	11.92093	160	0.37	FLUXNET2015	51
**US-GLE**	GLEES	ENF	Dfc	41.36653	-106.2399	3197	0.83	FLUXNET2015	52
**US-Mpj**	Mountainair Pinyon-Juniper Woodland	WSA	Bsk	34.4385	-106.2377	2196	0.29	OneFlux	45

(c) Eddy covariance sites with 5+ years of data in spring, summer, and fall (our minimum for inclusion in the study, n = 112), includes all previously listed sites, in addition to 48 more sites:

Site ID	Site Name	IGBP	Clim	Latitude	Longitude	Elev	WI	Data Source	Ref
**BE-Dor**	Dorinne	GRA	Cfb	50.31135	4.968591	247	1.31	ICOS-2020	53
**CA-Ca1**	British Columbia - 1949 Douglas-fir stand	ENF	Cfb	49.8673	-125.3336	300	2.60	OneFlux	54
**CA-Ca2**	British Columbia - Clearcut Douglas-fir stand (harvested winter 1999/2000)	ENF	Cfb	49.8705	-125.2909	300	2.28	OneFlux	54
**CA-Ca3**	British Columbia - Pole sapling Douglas-fir stand	ENF	Cfb	49.5346	-124.9004	120	2.26	OneFlux	54
**CA-Qfo**	Quebec - Eastern Boreal, Mature Black Spruce	ENF	Dfc	49.6925	-74.34206	382	2.06	FLUXNET2015	55
**CA-TP1**	Ontario - Turkey Point 2002 Plantation White Pine	ENF	Dfb	42.66093611	-80.55951944	265	1.33	FLUXNET2015	7
**CA-WP1**	Alberta - Western Peatland - LaBiche River,Black Spruce/Larch Fen	WET	Dfc	54.9538	-112.467	540	0.76	OneFlux	56
**CH-Aws**	Alp Weissenstein	GRA	Dfc	46.583306	9.790417	1988	3.17	ICOS-2020	57
**CH-Oe1**	Oensingen grassland	GRA	Cfb	47.28583	7.73194	450	1.69	FLUXNET2015	58
**CZ-Lnz**	Lanzhot	DBF	Cfb	48.68155	16.946331	150	0.82	ICOS-2020	59
**CZ-RAJ**	Rajec	ENF	Cfb	49.4437236	16.6965125	649	0.90	ICOS-2020	60
**DE-HoH**	Hohes Holz	DBF	Cfb	52.08656	11.22235	193	0.86	ICOS-2020	61
**DE-Hte**	Huetelmoor	WET	Cfb	54.210278	12.176111	1	1.02	ICOS-2018	62
**DE-Hzd**	Hetzdorf	DBF	Cfb	50.96381	13.48978	395	1.17	ICOS-2020	63
**DE-Lnf**	Leinefelde	DBF	Cfb	51.32822	10.3678	451	1.37	FLUXNET2015	64
**DE-RuR**	Rollesbroich	GRA	Cfb	50.62191	6.30413	514.7	1.79	ICOS-2020	65

**DE-RuW**	Wustebach	ENF	Cfb	50.50493	6.3309627	610	2.06	ICOS-2020	[66]
**ES-Abr**	Albuera	WSA	Csa	38.701839	-6.785881	279	0.42	ICOS-2020	[67]
**ES-LM1**	Majadas del Tietar North	WSA	Csa	39.94269	-5.778683	266	0.38	ICOS-2020	[68]
**ES-LM2**	Majadas del Tietar South	WSA	Csa	39.934592	-5.775881	270	0.38	ICOS-2020	[68]
**FI-Var**	Varrio	ENF	Dfc	67.7549	29.61	395	1.87	ICOS-2020	[69]
**FR-Bil**	Bilos	ENF	Cfb	44.493652	-0.956092	39	1.03	ICOS-2020	[70]
**FR-Hes**	Hesse	DBF	Cfb	48.6741	7.06465	310	1.12	ICOS-2020	[71]
**IT-Cpz**	Castelporziano	EBF	Csa	41.70525	12.37611	68	0.71	FLUXNET2015	[72]
**IT-Lsn**	Lison	OSH	Cfa	45.740482	12.750297	1	1.18	ICOS-2020	[73]
**IT-Ro1**	Roccarespampani 1	DBF	Csa	42.40812	11.93001	235	0.37	FLUXNET2015	[74]
**IT-SR2**	San Rossore 2	ENF	Csa	43.73202	10.29091	4	0.83	ICOS-2020	[75]
**NL-Hor**	Horstermeer	GRA	Cfb	52.24035	5.0713	2.2	1.36	FLUXNET2015	[76]
**RU-Fy2**	Fyodorovskoye dry spruce	ENF	Dfb	56.4476	32.9019	276	1.38	ICOS-2020	[36]
**SE-Htm**	Hyltemossa	ENF	Cfb	56.09763	13.41897	115	1.69	ICOS-2020	[77]
**SE-Nor**	Norunda	ENF	Dfb	60.0865	17.479504	45	1.19	ICOS-2020	[77]
**SE-Ros**	Rosinedal-3	ENF	Dfc	64.1725	19.738	157	1.56	ICOS-2020	[78]
**SE-Svb**	Svartberget	ENF	Dfc	64.25611	19.7745	267	1.65	ICOS-2020	[79]
**US-Blo**	Blodgett Forest	ENF	Csa	38.8953	-120.6328	1315	1.24	FLUXNET2015	[80]
**US-Dk2**	Duke Forest-hardwoods	DBF	Cfa	35.9736	-79.1004	168	1.05	OneFlux	[81]
**US-Dk3**	Duke Forest - loblolly pine	ENF	Cfa	35.9782	-79.0942	163	1.05	OneFlux	[82]
**US-Fmf**	Flagstaff - Managed Forest	ENF	Csb	35.1426	-111.7273	2160	0.48	OneFlux	[83]
**US-Fuf**	Flagstaff - Unmanaged Forest	ENF	Csb	35.089	-111.762	2180	0.50	OneFlux	[83]
**US-Ho1**	Howland Forest (main tower)	ENF	Dfb	45.2041	-68.7402	60	1.64	OneFlux	[84]
**US-IB2**	Fermi National Accelerator Laboratory- Batavia (Prairie site)	GRA	Dfa	41.84062	-88.24103	226.5	1.08	FLUXNET2015	[85]
**US-KFS**	Kansas Field Station	GRA	Cfa	39.0561	-95.1907	310	0.91	OneFlux	[86]
**US-Los**	Lost Creek	WET	Dfb	46.0827	-89.9792	480	1.31	FLUXNET2015	[50]
**US-Me3**	Metolius-second young aged pine	ENF	Csb	44.3154	-121.6078	1005	0.55	FLUXNET2015	[87]
**US-Prr**	Poker Flat Research Range Black Spruce Forest	ENF	Dwc	65.12367	-147.48756	210	0.76	OneFlux	[88]
**US-SRC**	Santa Rita Creosote	OSH	Bwh	31.9083	-110.8395	950	0.21	FLUXNET2015	[89]
**US-Syv**	Sylvania Wilderness Area	MF	Dfb	46.242	-89.3477	540	1.29	FLUXNET2015	[90]
**US-Tw1**	Twitchell Wetland West Pond	WET	Csa	38.1074	-121.6469	-5	0.29	OneFlux	[91]
**US-Whs**	Walnut Gulch Lucky Hills Shrub	OSH	Bsk	31.7438	-110.0522	1370	0.20	FLUXNET2015	[46]

Supplemental Table S2: Terrestrial biosphere models from TRENDY v6 used in analysis. Acronyms are from 'TBM' labels in Figure 2 and citations from Le Quéré et al. (2018)[92] and Sun et al. (2021)[93].

**TBM**	**Reference**
CABLE	Haverd et al. (2017)[94]
CLASS-CTEM	Melton and Arora (2016)[95]
CLM	Oleson et al. (2013)[96]
DLEM	Tian et al. (2015)[97]
ISAM	Jain et al. (2013)[98]
JSBACH	Reick et al. (2013)[99]
JULES	Clark et al. (2011)[100]
LPJ	Sitch et al. (2003)[101]
LPJ-GUESS	Smith et al. (2014)[102]
LPX	Keller et al. (2017)[103]
ORCHIDEE	Krinner et al. (2005)[104]
ORCHIDEE-MICT	Guimberteau et al. (2018)[105]
VEGAS	Zeng et al. (2005)[106]
VISIT	Kato et al. (2013)[107]

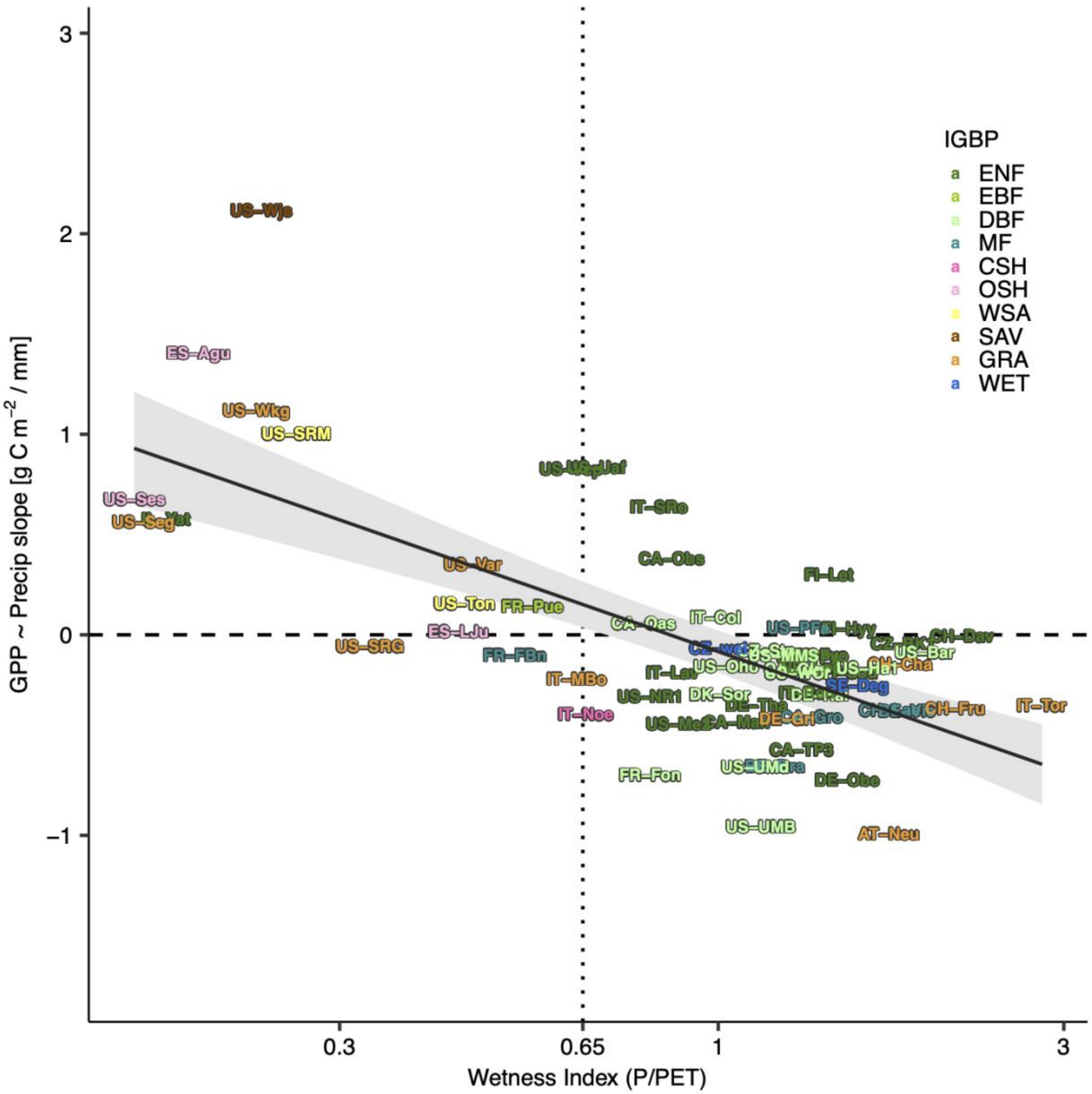

Supplemental Figure S2: Eddy covariance site IDs for spring GPP sensitivity to precipitation. Same as Figure 1a in main text but with site IDs in (a).

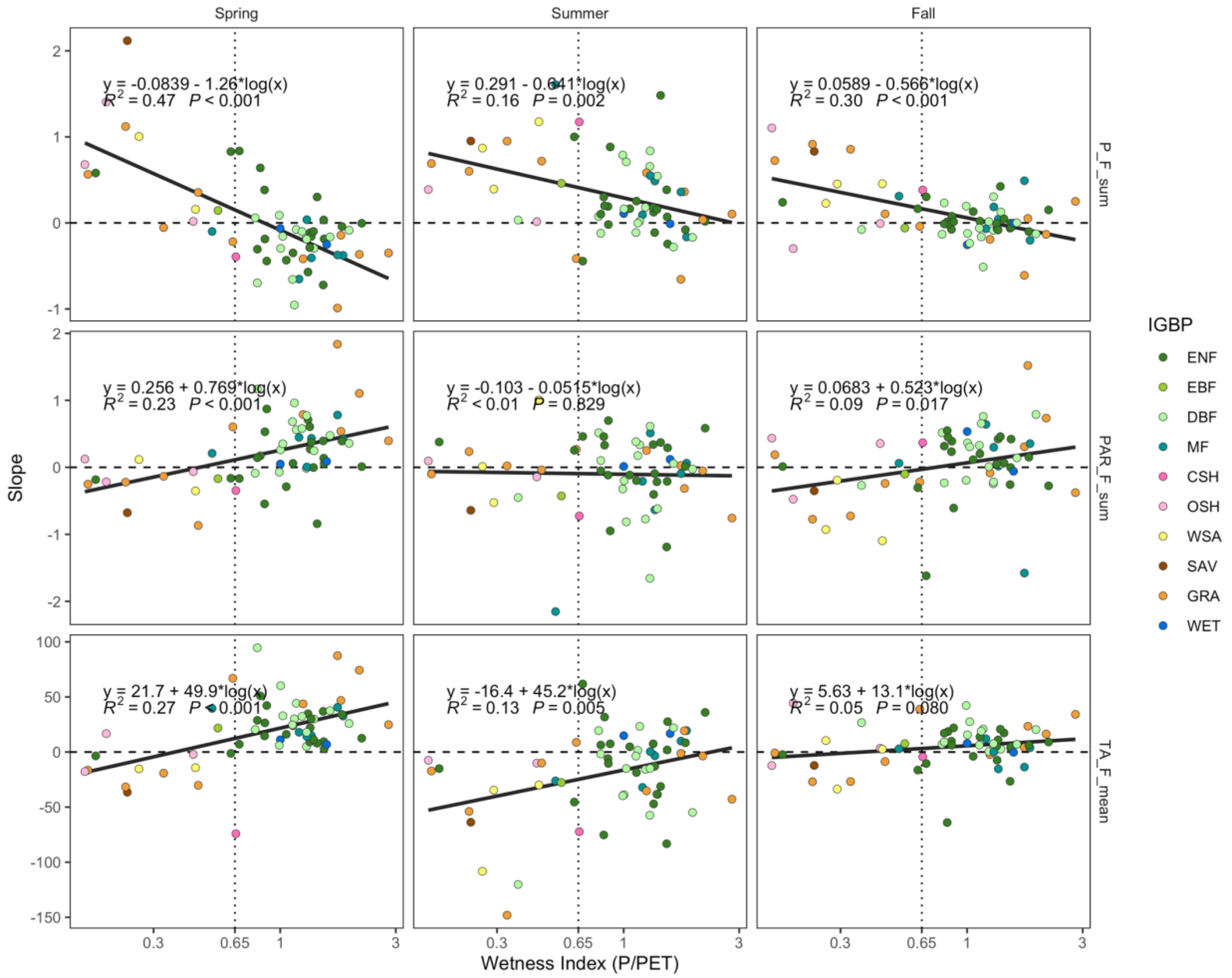

Supplemental Figure S3: GPP sensitivity to precipitation, PAR, and air temperature in different seasons for sites with 10+ years of data per season. Columns are spring (61 sites), summer (62 sites), and fall (63 sites); rows are precipitation (g C m^{-2} / mm), PAR (g C / MJ), and air temperature (g C m^{-2} / °C) regressions. The site IL-Yat is excluded in the regression for summer GPP~precipitation (top center) due to its outlying extreme sensitivity at 23.3 g C m^{-2} / mm; if it is included, the regression is: $y = 0.498 – 3.58*\log_{10}(x)$, $R^2 = 0.13$, $p = 0.004$.

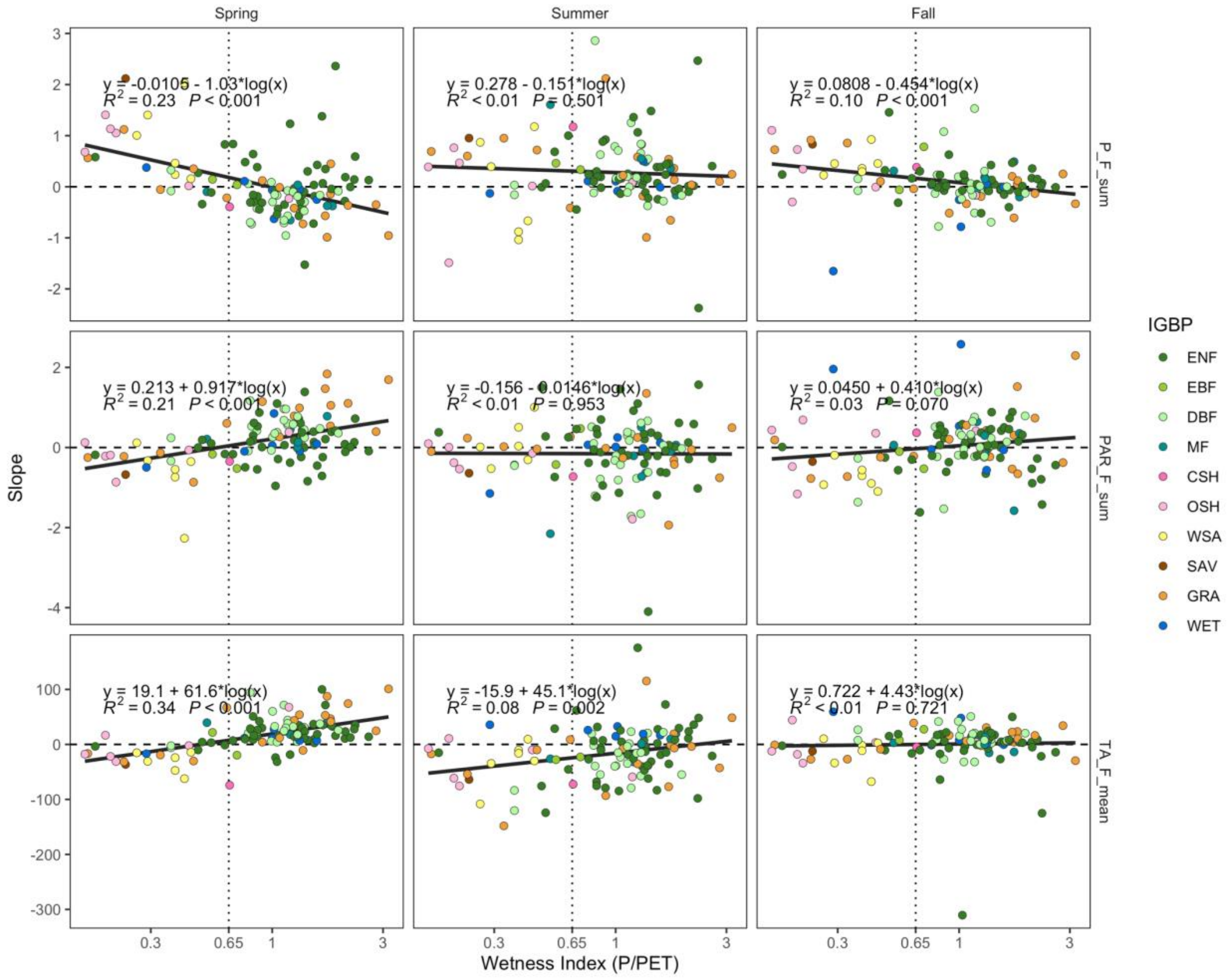

Supplemental Figure S4: GPP sensitivity to precipitation, PAR, and air temperature in different seasons for sites with 5+ years of data per season. Columns are spring, summer, and fall (112 sites for all seasons); rows are precipitation (g C m^{-2} / mm), PAR (g C / MJ), and air temperature (g C m^{-2} / °C) regressions. The site IL-Yat is excluded in the regression for summer GPP~precipitation (top center) due to its outlying extreme sensitivity at 23.3 g C m^{-2} / mm; if it is included, the regression is: $y = 0.423 – 1.88*\log_{10}(x)$, $R^2 = 0.06$, $p = 0.01$.

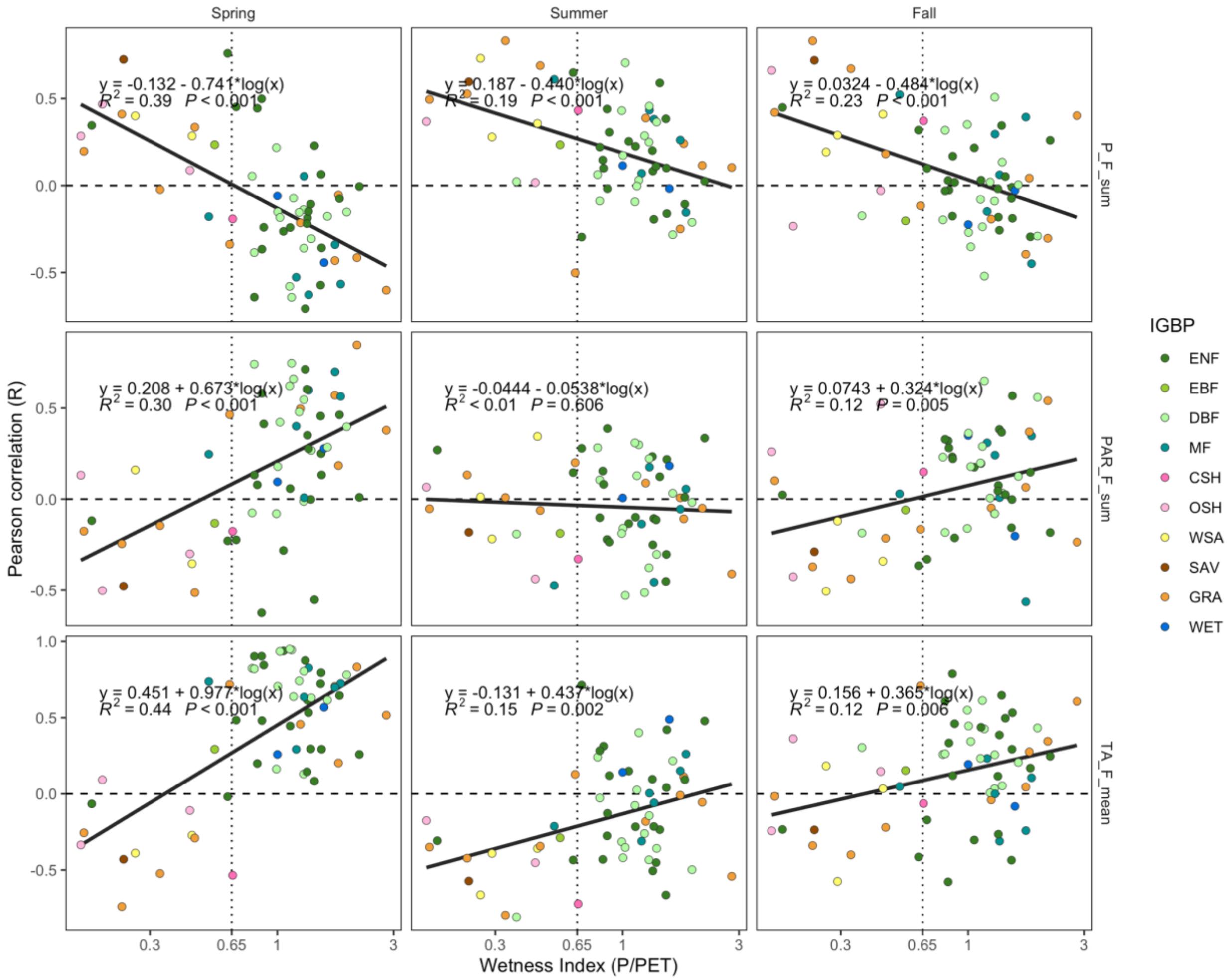

Supplemental Figure S5: Pearson correlations for GPP sensitivities for all sites with 10+ years of data per season. Precipitation, PAR, and air temperature in different seasons for sites.

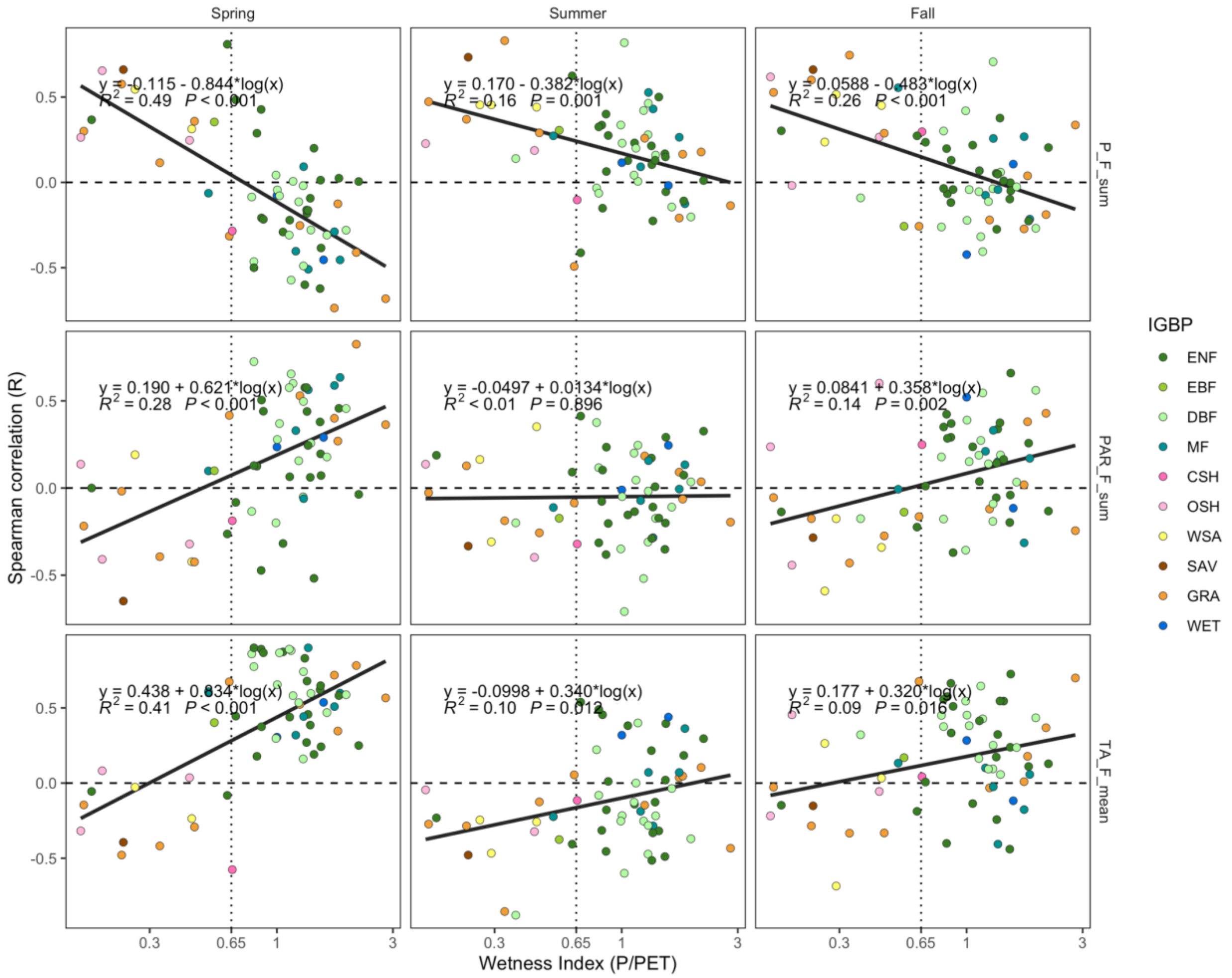

Supplemental Figure S6: Spearman correlations for GPP sensitivities for all sites with 10+ years of data per season. Precipitation, PAR, and air temperature in different seasons.

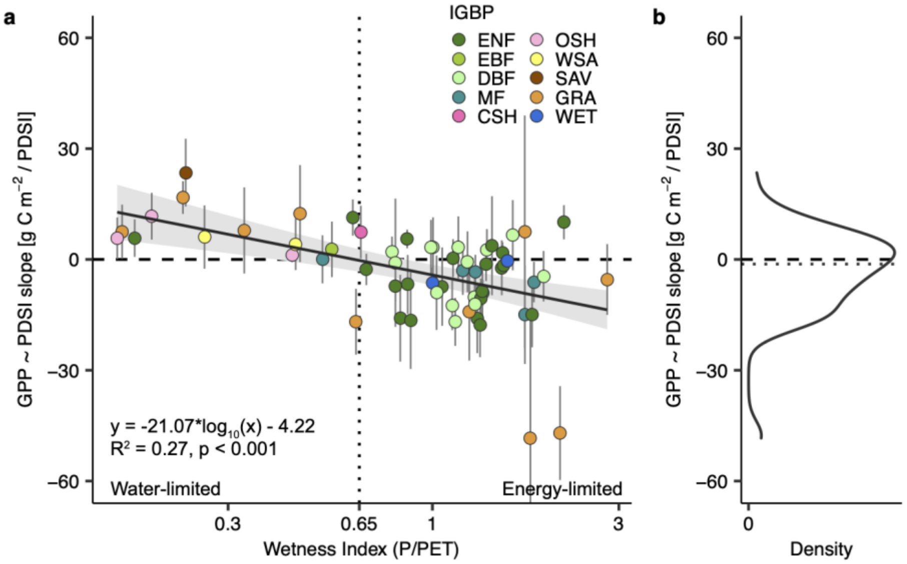

Supplemental Figure S7: GPP sensitivity to TerraClimate PDSI during the spring. Following Figure 1, (a) sensitivity of each site along aridity gradient and (b) density of site sensitivities. 14/15 = 93% of the water-limited sites having positive GPP~PDSI relationships, and 33/46 = 72% of the energy-limited sites having negative GPP~PDSI relationships.

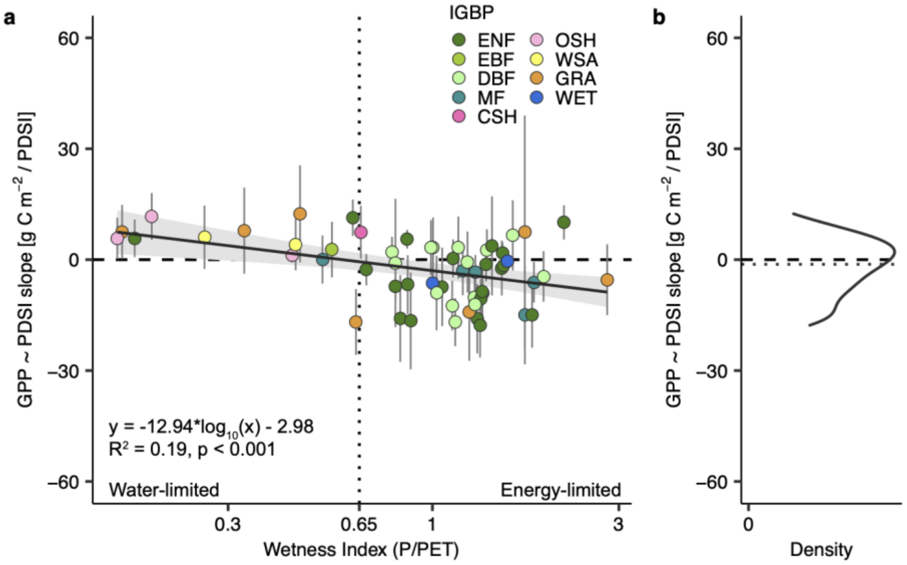

Supplemental Figure S8: GPP sensitivity to TerraClimate PDSI during the spring, removing outliers by retaining only the middle 95% of site sensitivities. Following Figure 1, (a) sensitivity of each site along aridity gradient and (b) density of site sensitivities. 12/13 = 92% of the water-limited sites having positive GPP~PDSI relationships, and 31/44 = 70% of the energy-limited sites having negative GPP~PDSI relationships.

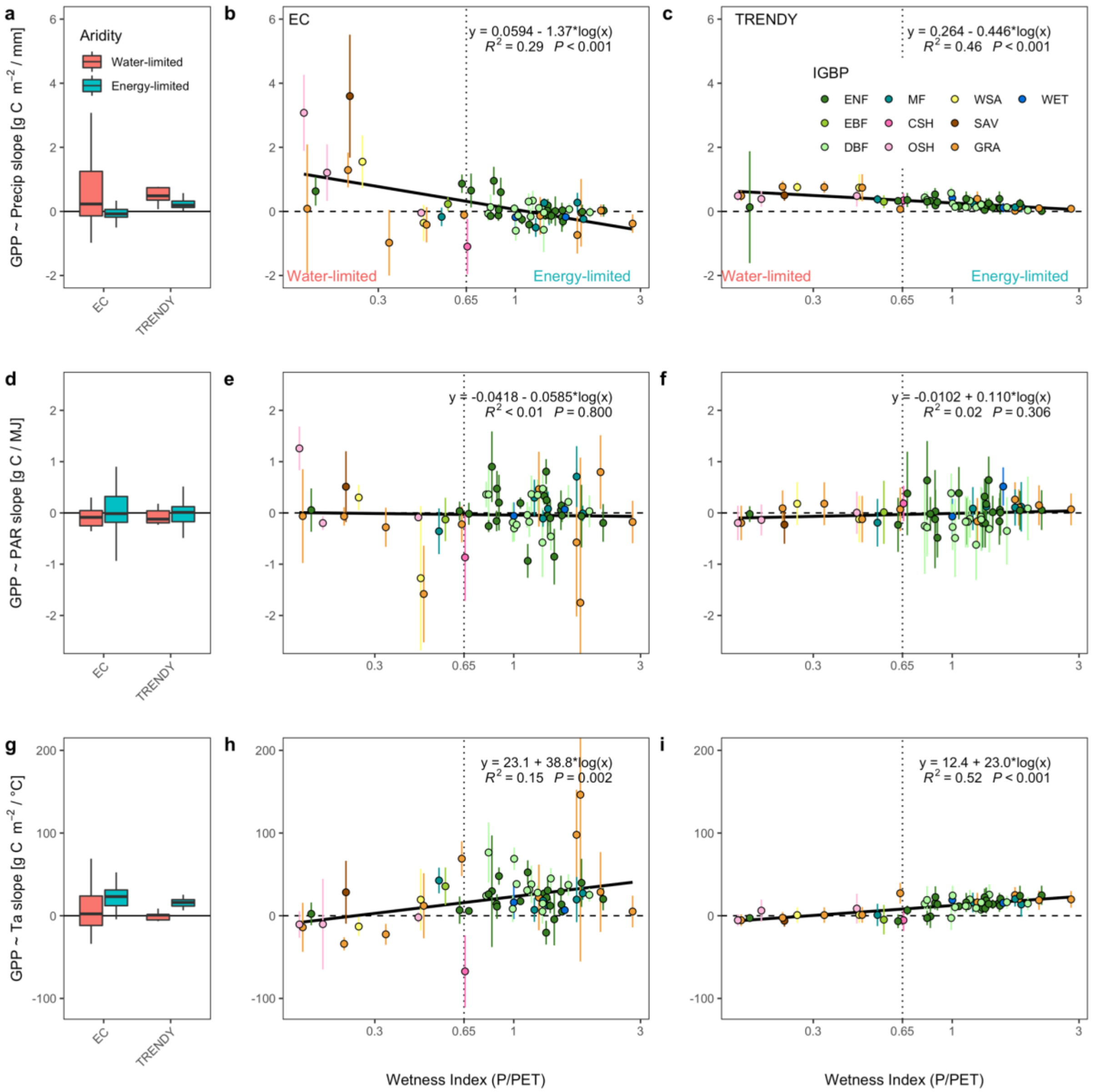

Supplemental Figure S9: GPP sensitivities from multiple linear regression during spring. Top is precipitation, middle is PAR, and bottom is air temperature. Left column is comparison of sensitivities for water-limited (dry) and energy-limited (wet) sites in EC and TRENDY model mean. Middle column is EC data, and right column is TRENDY model mean. In both the EC and TRENDY model mean data, the partial GPP sensitivity to precipitation during the spring vs. WI remains significant (EC: **b**; TRENDY: **c**) as does the partial GPP sensitivity to air temperature (EC: **h**, TRENDY: **i**). PAR provides no additional information towards GPP sensitivity along the aridity gradient for either EC or TRENDY model mean when precipitation and air temperature are accounted for (**e, f**). This is likely due to the strong correlation between the univariate sensitivity of GPP to PAR and the univariate sensitivity of GPP to air temperature across sites (R^2 = 0.57, **Supplemental Figure S20**). The Spearman correlations between GPP and PAR and GPP and air temperature are positively correlated, except for the most temperature-controlled sites, which are likely too cold to consistently benefit from extra PAR (**Supplemental Figure S21**).

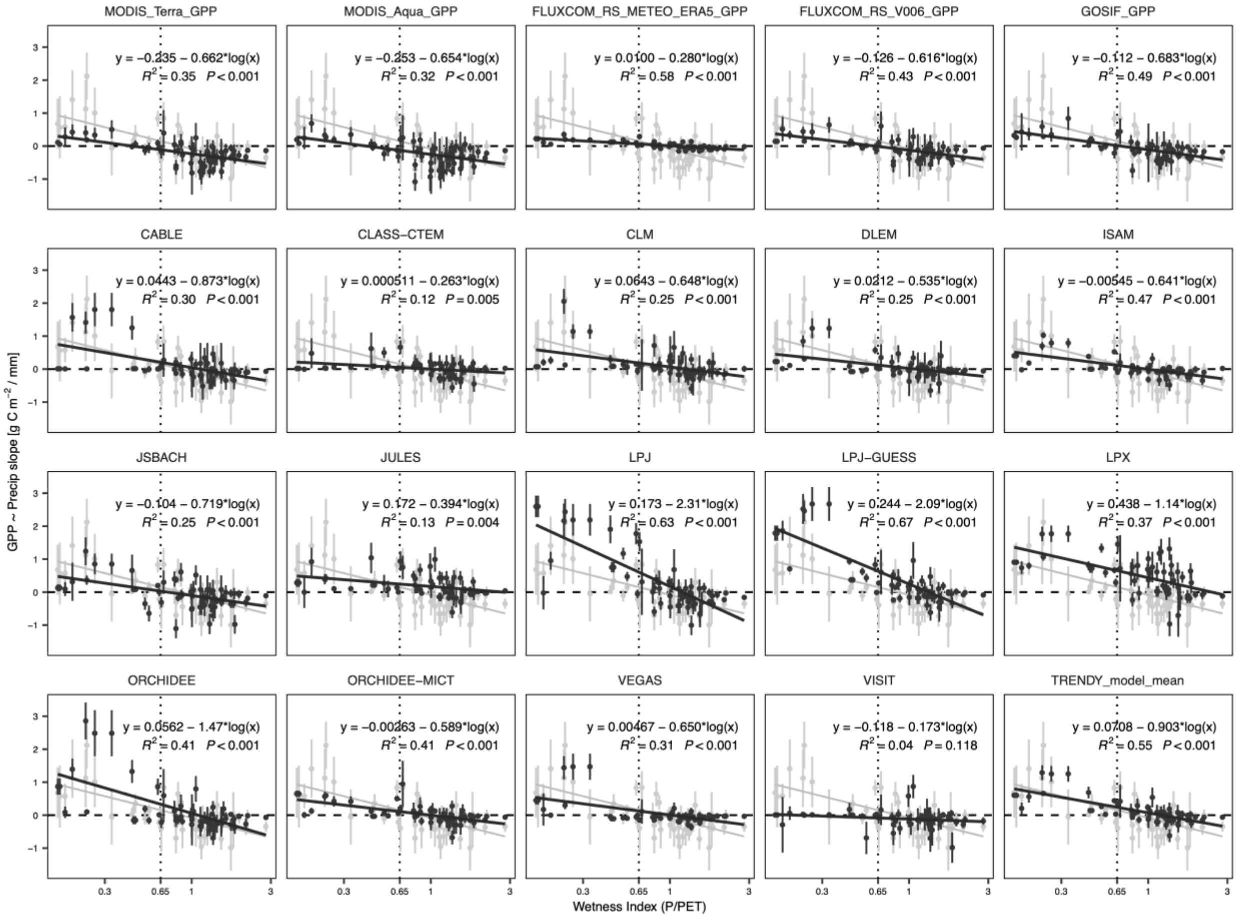

Supplemental Figure S10: Spring GPP sensitivity to precipitation (following Figure 1) for all TRENDY models and remote sensing GPP data sets. Individual models and remote sensing datasets are black dots and lines. EC data from Figure 1 are gray dots and lines underneath each plot. Water-limited sites are to the left of the vertical dotted line (x = 0.65), and energy-limited sites are to the right. Equations on plot are for black points and lines; EC equation from Figure 1 (for gray lines) is $1.26*\log_{10}x - 0.08$; $R^2 = 0.47$, $p < 0.001$.

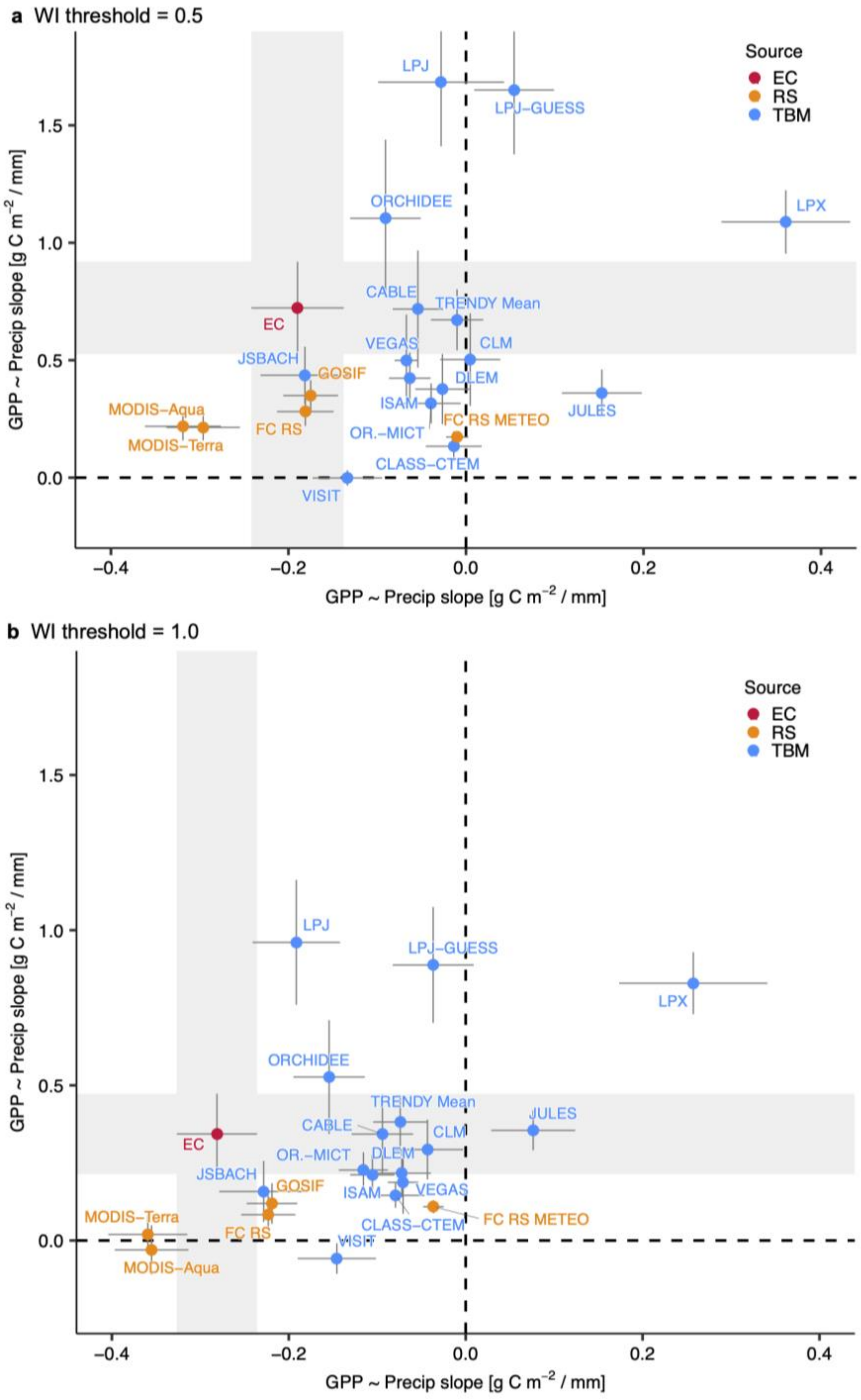

Supplemental Figure S11: Even when changing WI thresholds, EC observations of spring GPP sensitivities to precipitation occupy a different region of sensitivity space than models and satellite products. Same as Figure 2 but changing the WI threshold to (a) 0.5 and (b) 1.0. (The version shown in Figure 2 in main text is listed at 0.65.) All axes are scaled such that 1 SE is the same size in both directions within each plot.

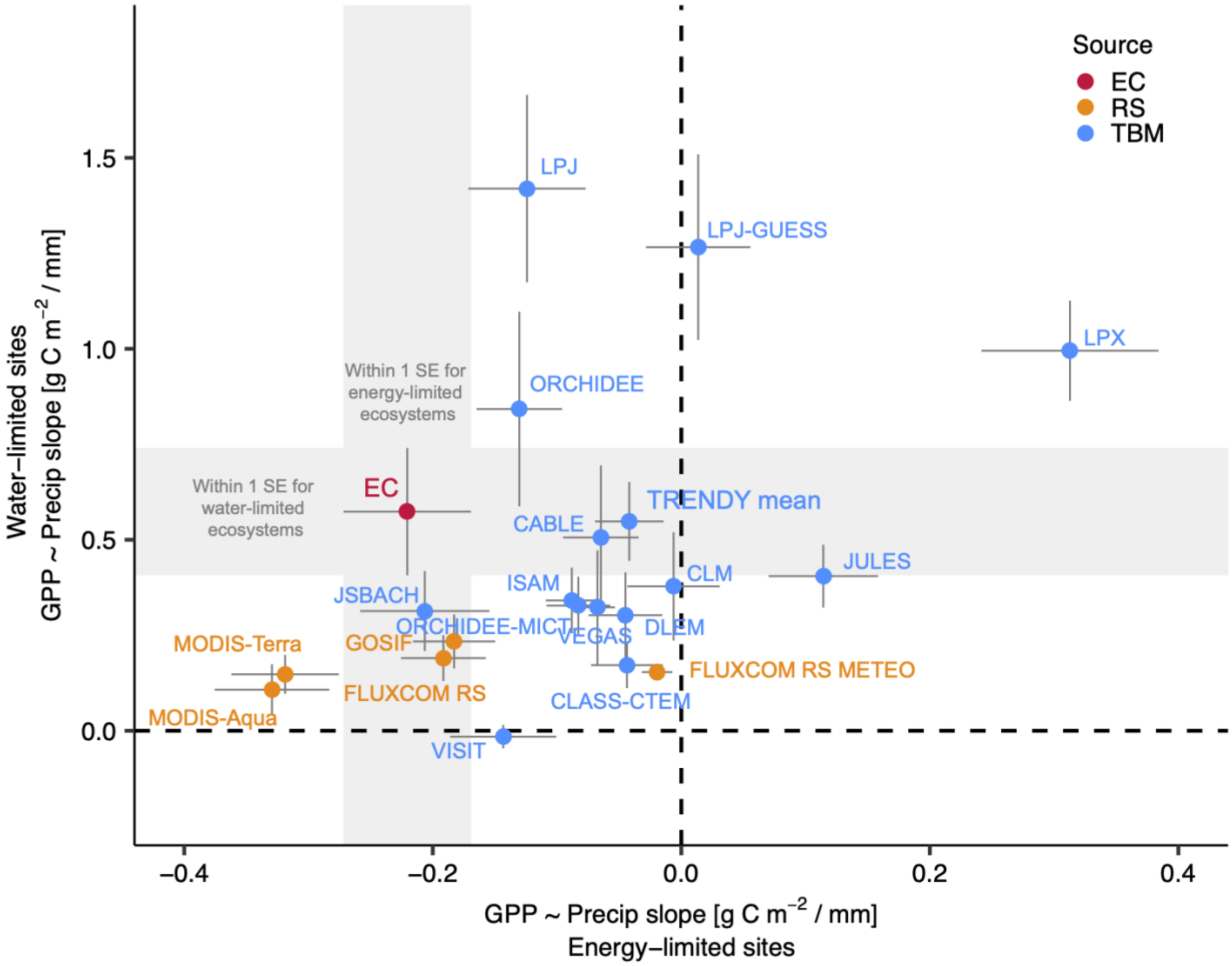

Supplemental Figure S12: Replacing WI from TBMs with CRU TS 4.05. Changing TBM WI input from TerraClimate to CRU TS 4.05 shifts some TBMs a small amount but does not change the overall conclusions from Figure 2 that the TBMs underestimate the negative sensitivity from energy-limited sites that is shown in the EC data.

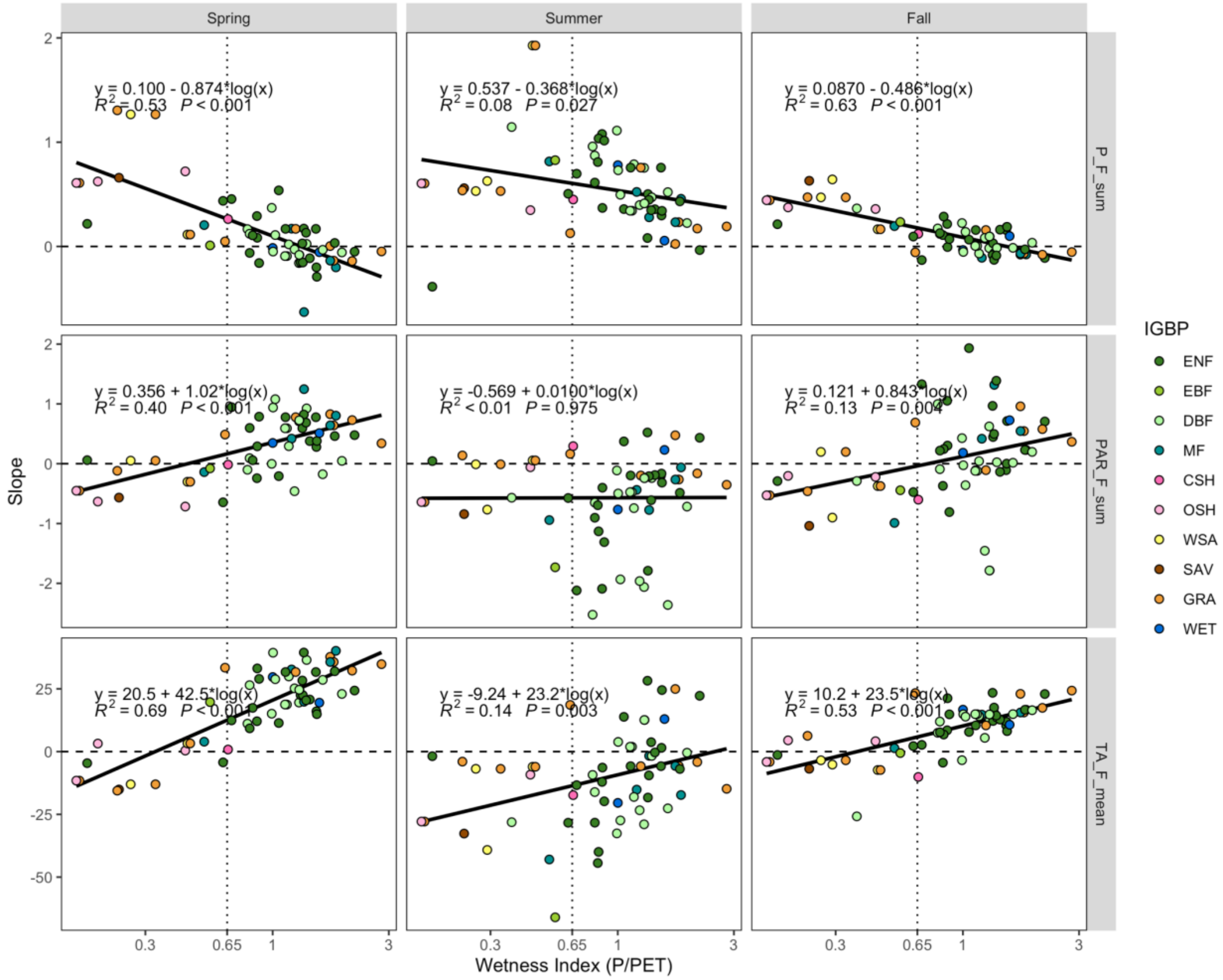

Supplemental Figure S13: Regressions for TRENDY model mean slopes for each meteorological variable's GPP sensitivity across WI for each season. (Same as Supplemental Figure S3, but for TRENDY model means.)

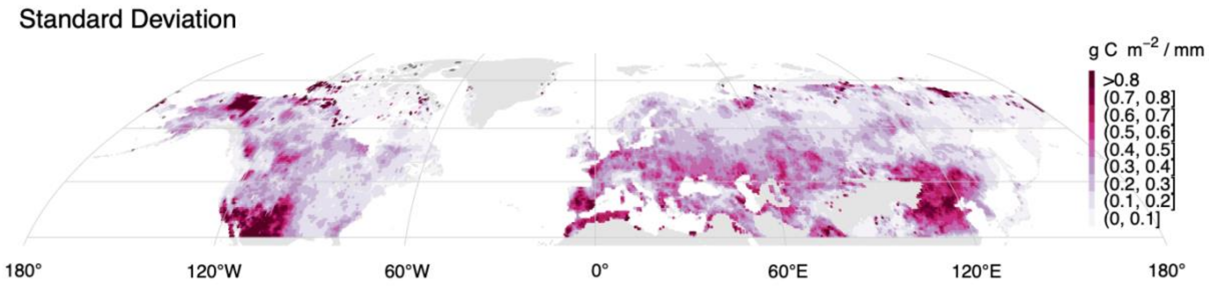

Supplemental Figure S14: Standard deviation in GPP~precipitation sensitivity across all included TRENDY models during the spring. Models are most consistent (lowest SD) in humid regions and least consistent (highest SD) in arid regions with some exceptions: north-central Europe has relatively high SD despite being humid, and western China has relatively low SD despite being arid. Base maps of the continents in are in the public domain from Natural Earth.

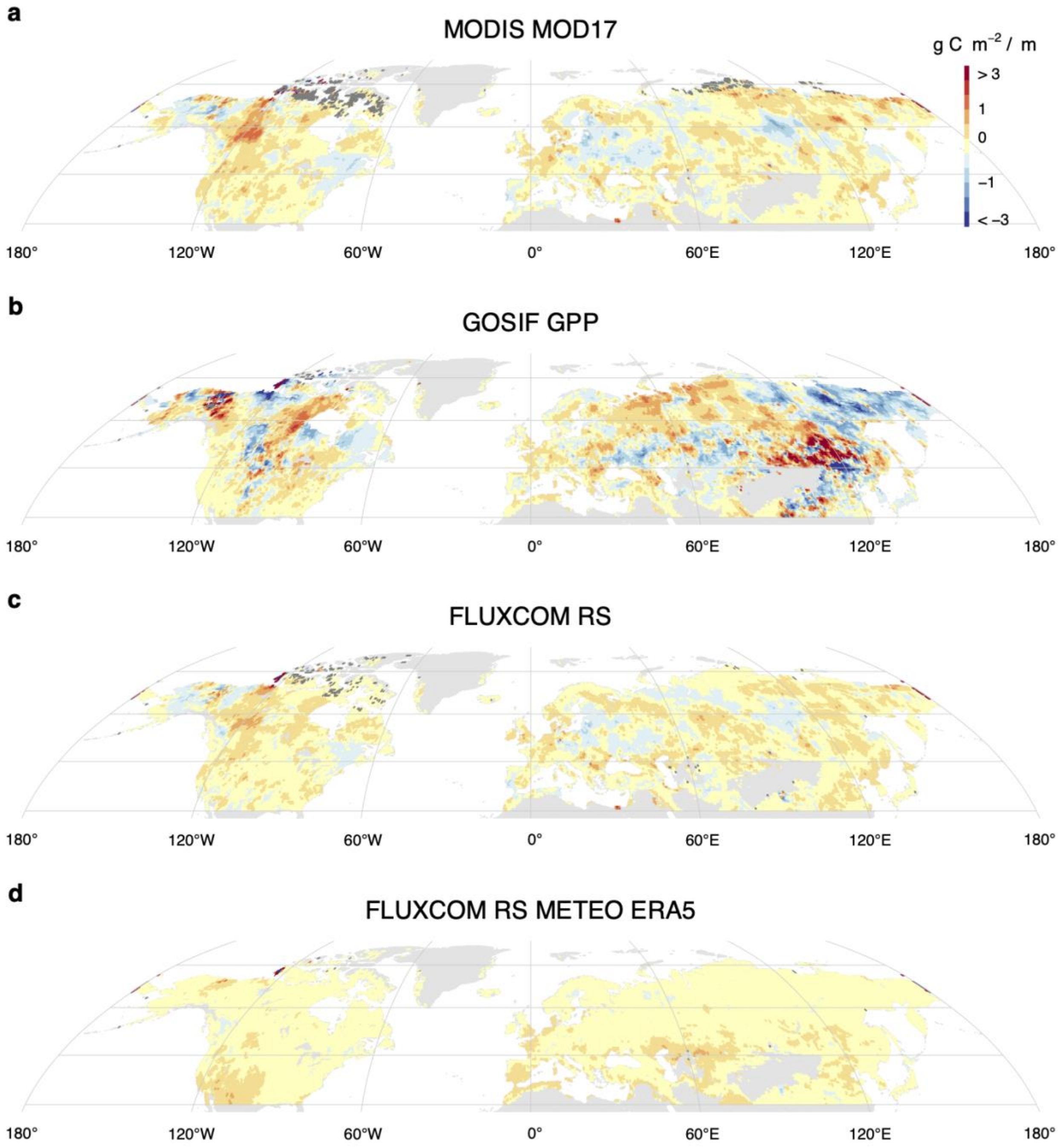

Supplemental Figure S15: Maps of sensitivity of GPP in remote sensing products to spring precipitation. Note that MODIS MOD17 is MODIS-Terra. Base maps of the continents in are in the public domain from Natural Earth.

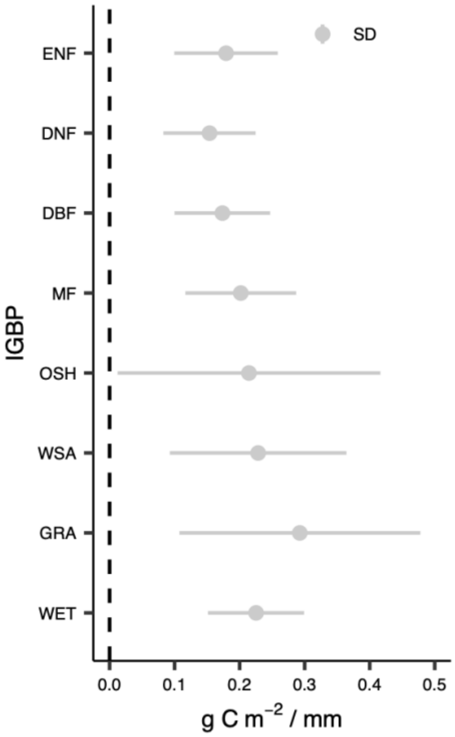

Supplemental Figure S16: Standard deviation across all included TRENDY models by biome. Grasslands (GRA) have the greatest variation in estimates across models. Other included biomes are: evergreen needleleaf forests (ENF), deciduous needleleaf forests (DNF), deciduous broadleaf forests (DBF), mixed forests (MF), open shrublands (OSH), woody savanna (WSA), and wetlands (WET).

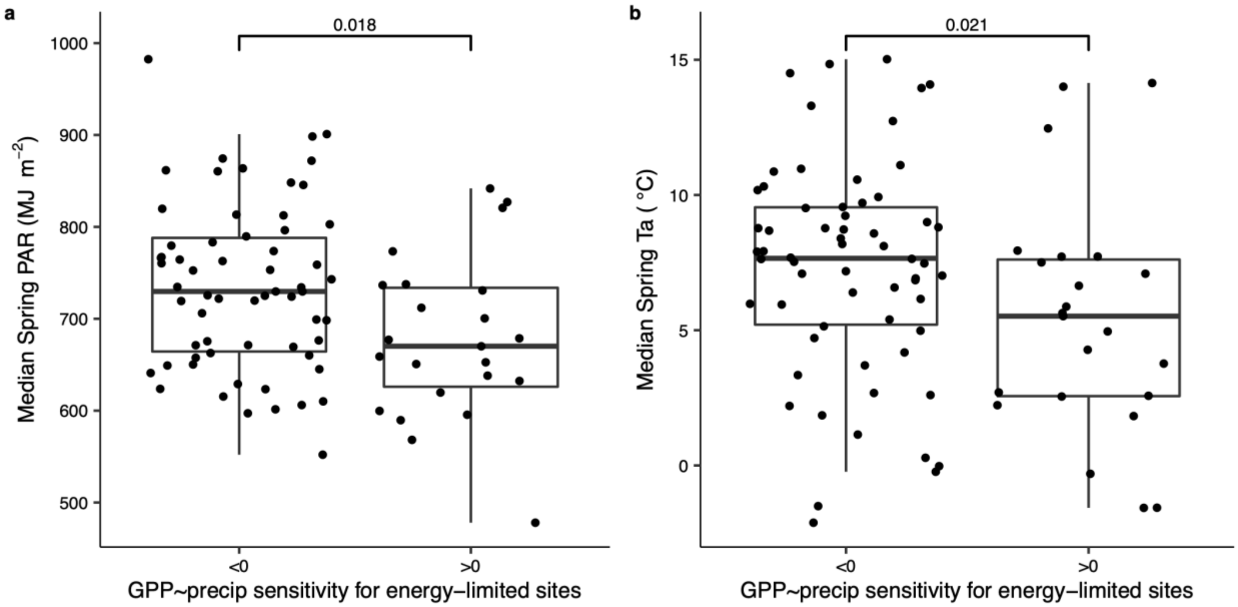

Supplemental Figure S17: Energy-limited sites with positive GPP~precip sensitivities in the spring tend to have significantly lower PAR and air temperature than energy-limited sites with negative sensitivities in the spring. Comparing energy-limited site GPP~precip sensitivities for sites with positive (>0) or negative (<0) sensitivities for (a) the sum of median spring PAR ($p = 0.018$) and (b) median spring air temperature ($p = 0.021$). Bars across the top indicate p-values, with both plots having significant differences at alpha = 0.05. Box has median with 25th and 75th percentiles, and lines extend to 1.5*IQR; all points have horizontal dodging for visual separation.

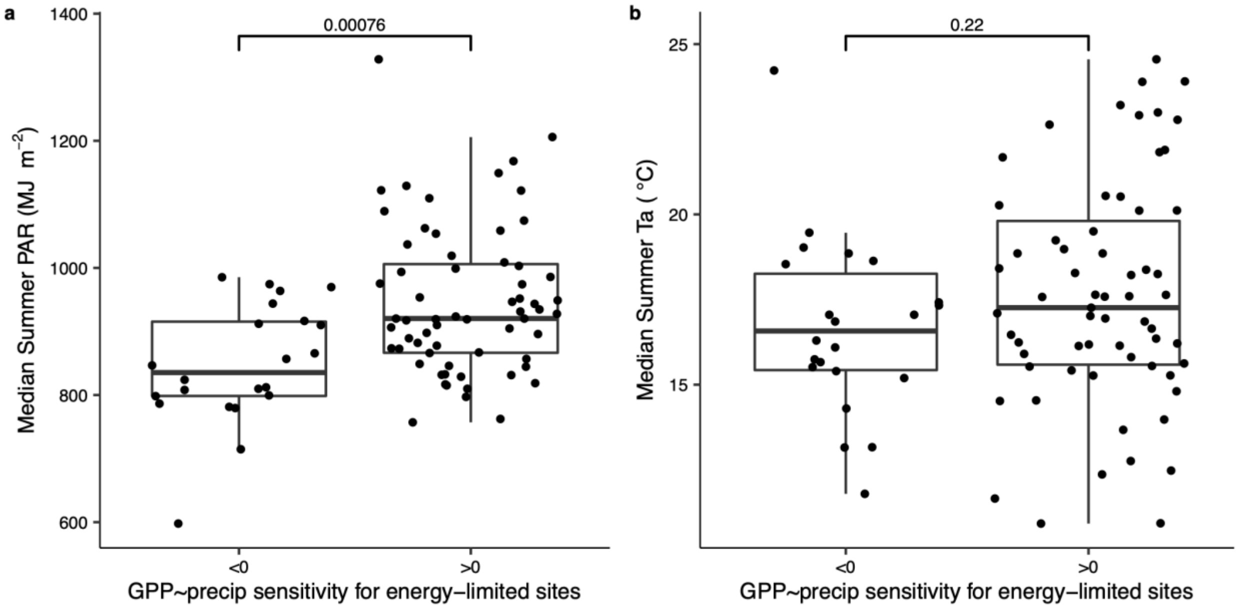

Supplemental Figure S18: Energy-limited sites that maintain negative GPP~precip sensitivities during the summer receive less solar radiation ((a) lower PAR, $p < 0.001$), but (b) are not necessarily colder ($p = 0.22$). Points are individual EC sites and bars across the top indicate p-values. Boxes are the same layout as previous plot.

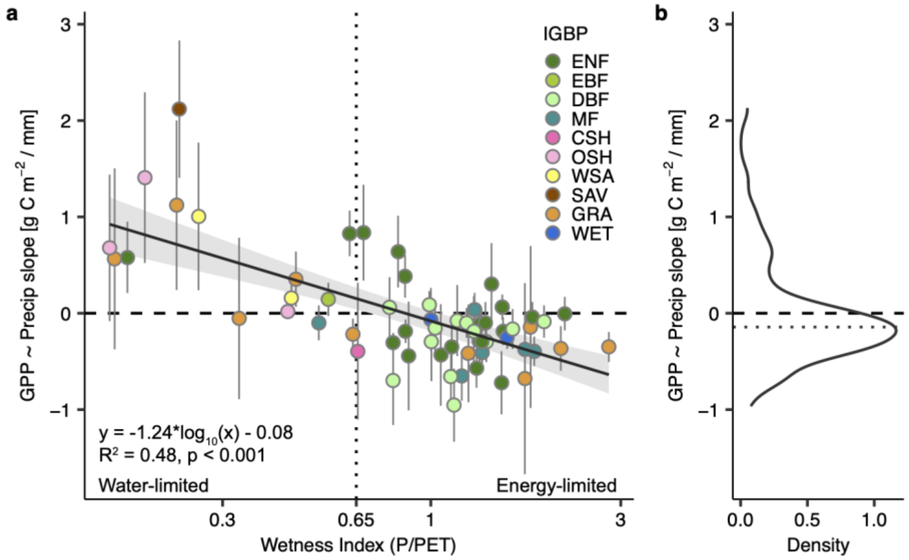

Supplemental Figure S19: Detrending precipitation for the GPP~precipitation slopes in spring produces nearly an identical regression to Figure 1. The only sites that change their slopes are AT-Neu, BE-Vie, FI-Hyy, and NL-Loo.

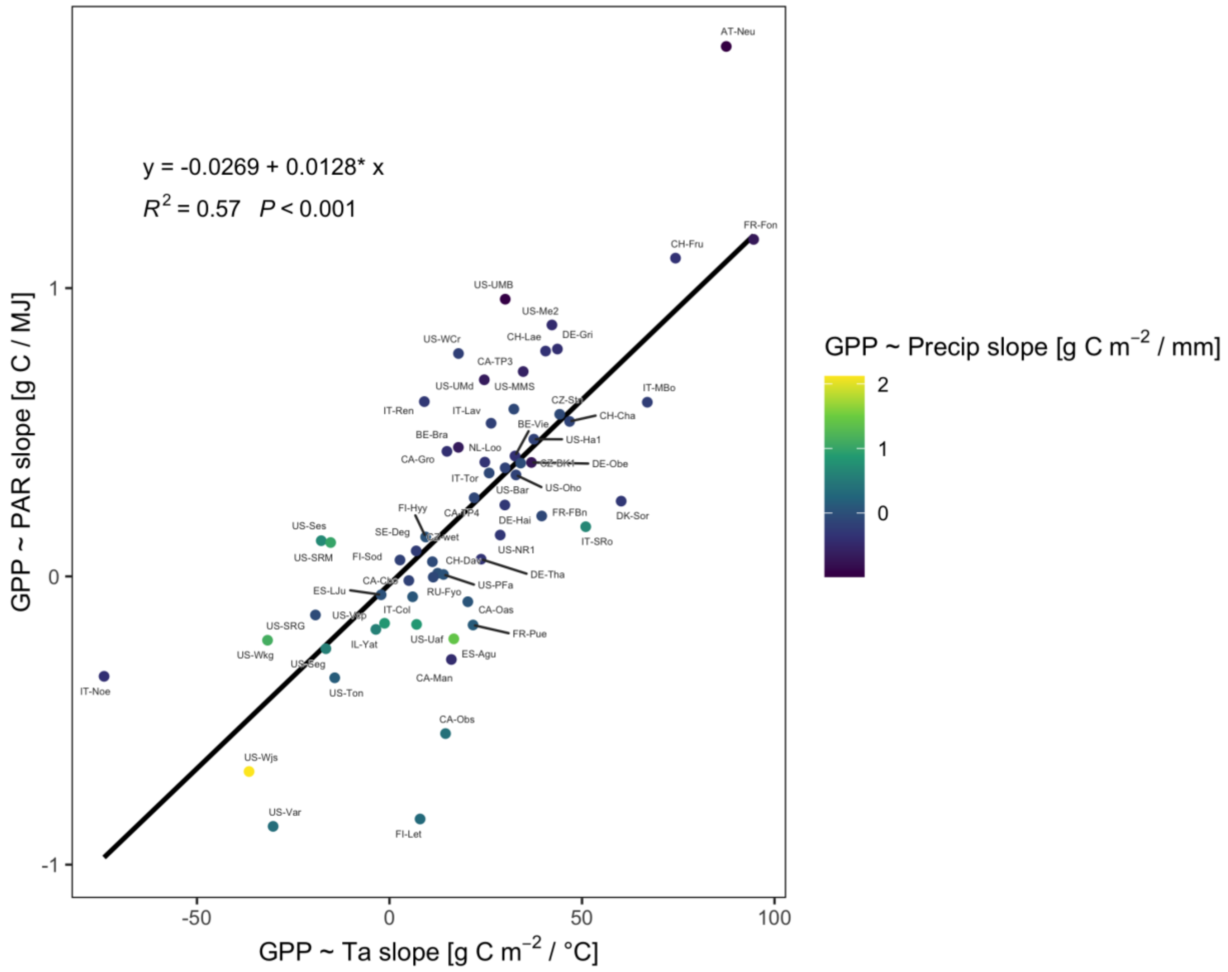

Supplemental Figure S20: Sensitivity of spring GPP to air temperature (x) and spring GPP to PAR (y) are strongly correlated across sites. Points are given site labels and colored by their spring GPP sensitivity to precipitation.

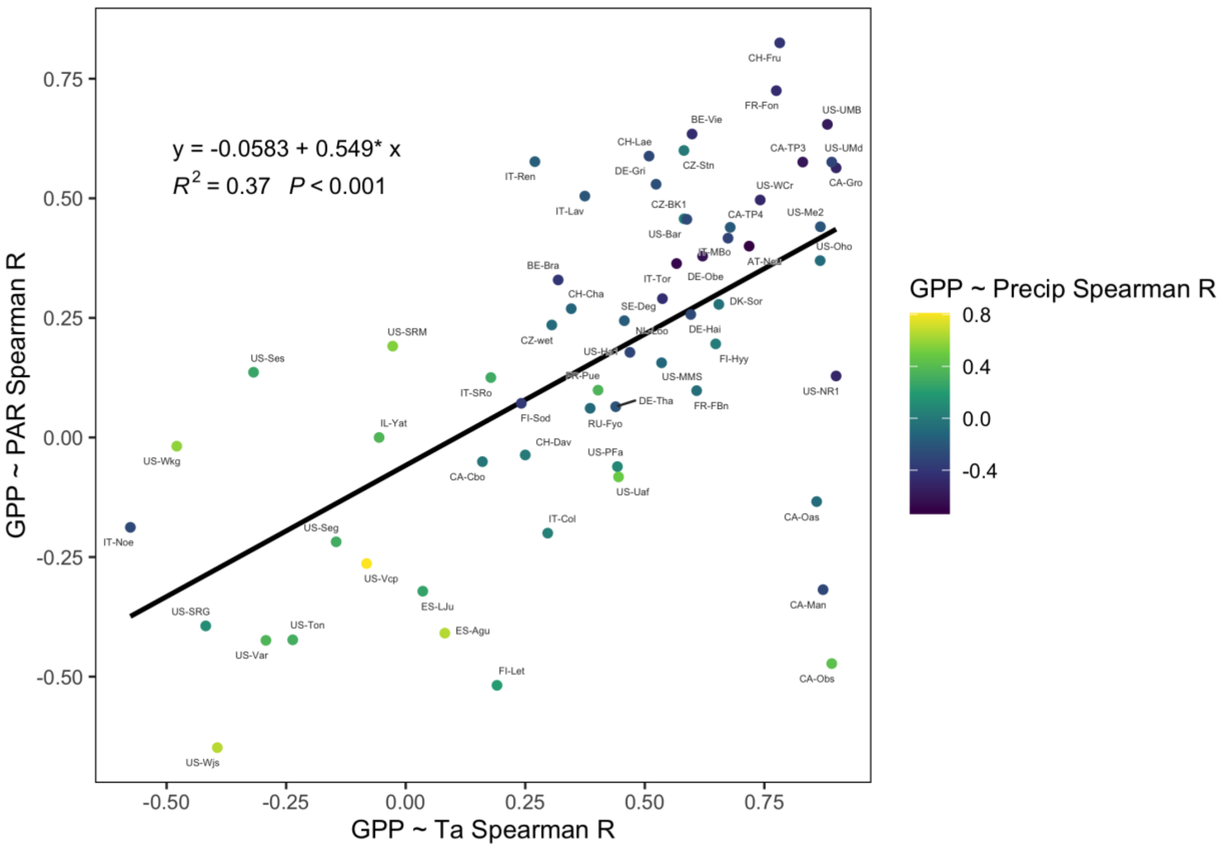

Supplemental Figure S21: Same as previous figure, but with Spearman ranked correlation instead of slopes. Notice the sites on the right with high GPP ~ Ta correlations that fall off the line (US-NR1, CA-Oas, CA-Man, CA-Obs) because these sites have very cold weather during spring.

References for Supplemental Material – Figures and Tables

1. Wohlfahrt, G. *et al.* Seasonal and inter-annual variability of the net ecosystem CO2 exchange of a temperate mountain grassland: Effects of weather and management. *J. Geophys. Res. Atmospheres* **113**, (2008).
2. Carrara, A., Janssens, I. A., Curiel Yuste, J. & Ceulemans, R. Seasonal changes in photosynthesis, respiration and NEE of a mixed temperate forest. *Agric. For. Meteorol.* **126**, 15–31 (2004).
3. Aubinet, M. *et al.* Long term carbon dioxide exchange above a mixed forest in the Belgian Ardennes. *Agric. For. Meteorol.* **108**, 293–315 (2001).
4. Lee, X., Fuentes, J. D., Staebler, R. M. & Neumann, H. H. Long-term observation of the atmospheric exchange of CO2 with a temperate deciduous forest in southern Ontario, Canada. *J. Geophys. Res. Atmospheres* **104**, 15975–15984 (1999).
5. McCaughey, J. H., Pejam, M. R., Arain, M. A. & Cameron, D. A. Carbon dioxide and energy fluxes from a boreal mixedwood forest ecosystem in Ontario, Canada. *Agric. For. Meteorol.* **140**, 79–96 (2006).
6. Margolis, H. A. & Ryan, M. G. A physiological basis for biosphere–atmosphere interactions in the boreal forest: an overview. *Tree Physiol.* **17**, 491–499 (1997).
7. Arain, M. A. & Restrepo-Coupe, N. Net ecosystem production in a temperate pine plantation in southeastern Canada. *Agric. For. Meteorol.* **128**, 223–241 (2005).
8. Merbold, L. *et al.* Greenhouse gas budget (CO2, CH4 and N2O) of intensively managed grassland following restoration. *Glob. Change Biol.* **20**, 1913–1928 (2014).
9. Zielis, S. *et al.* NEP of a Swiss subalpine forest is significantly driven not only by current but also by previous year's weather. *Biogeosciences* **11**, 1627–1635 (2014).
10. Imer, D., Merbold, L., Eugster, W. & Buchmann, N. Temporal and spatial variations of soil CO2, CH4 and N2O fluxes at three differently managed grasslands. *Biogeosciences* **10**, 5931–5945 (2013).
11. Etzold, S. *et al.* The Carbon Balance of Two Contrasting Mountain Forest Ecosystems in Switzerland: Similar Annual Trends, but Seasonal Differences. *Ecosystems* **14**, 1289–1309 (2011).
12. Acosta, M. *et al.* Soil surface CO2 efflux measurements in Norway spruce forests: Comparison between four different sites across Europe — from boreal to alpine forest. *Geoderma* **192**, 295–303 (2013).
13. Mensah, C. *et al.* Environmental Effects on Normalized Gross Primary Productivity in Beech and Norway Spruce Forests. *Atmosphere* **12**, 1128 (2021).
14. Dušek, J., Čížková, H., Stellner, S., Czerný, R. & Květ, J. Fluctuating water table affects gross ecosystem production and gross radiation use efficiency in a sedge-grass marsh. *Hydrobiologia* **692**, 57–66 (2012).
15. Prescher, A. K., Grünwald, T. & Bernhofer, C. Land use regulates carbon budgets in eastern Germany: From NEE to NBP. *Agric. For. Meteorol.* **150**, 1016–1025 (2010).
16. Knohl, A., Schulze, E. D., Kolle, O. & Buchmann, N. Large carbon uptake by an unmanaged 250-year-old deciduous forest in Central Germany. *Agric. For. Meteorol.* **118**, 151–167 (2003).
17. Grünwald, T. & Bernhofer, C. A decade of carbon, water and energy flux measurements of an old spruce forest at the Anchor Station Tharandt. *Tellus B Chem. Phys. Meteorol.* **59**, 387–396 (2007).

18. Pilegaard, K., Ibrom, A., Courtney, M. S., Hummelshøj, P. & Jensen, N. O. Increasing net CO2 uptake by a Danish beech forest during the period from 1996 to 2009. *Agric. For. Meteorol.* **151**, 934–946 (2011).
19. Rey, A. *et al.* Wind as a main driver of the net ecosystem carbon balance of a semiarid Mediterranean steppe in the South East of Spain. *Glob. Change Biol.* **18**, 539–554 (2012).
20. Serrano-Ortiz, P. *et al.* Interannual CO2 exchange of a sparse Mediterranean shrubland on a carbonaceous substrate. *J. Geophys. Res. Biogeosciences* **114**, (2009).
21. Suni, T. *et al.* Long-term measurements of surface fluxes above a Scots pine forest in Hyytiälä, southern Finland, 1996-2001. *Boreal Environ. Res.* **8**, 287–301 (2003).
22. Koskinen, M. *et al.* Measurements of CO_2 exchange with an automated chamber system throughout the year: challenges in measuring night-time respiration on porous peat soil. *Biogeosciences* **11**, 347–363 (2014).
23. Thum, T. *et al.* Parametrization of two photosynthesis models at the canopy scale in a northern boreal Scots pine forest. *Tellus Ser. B-Chem. Phys. Meteorol.* **59**, 874–890 (2007).
24. Pisek, J. *et al.* Retrieval and validation of forest background reflectivity from daily Moderate Resolution Imaging Spectroradiometer (MODIS) bidirectional reflectance distribution function (BRDF) data across European forests. *Biogeosciences* **18**, 621–635 (2021).
25. Delpierre, N., Berveiller, D., Granda, E. & Dufrêne, E. Wood phenology, not carbon input, controls the interannual variability of wood growth in a temperate oak forest. *New Phytol.* **210**, 459–470 (2016).
26. Rambal, S., Joffre, R., Ourcival, J. M., Cavender-Bares, J. & Rocheteau, A. The growth respiration component in eddy CO2 flux from a Quercus ilex mediterranean forest. *Glob. Change Biol.* **10**, 1460–1469 (2004).
27. Grünzweig, J. M., Lin, T., Rotenberg, E., Schwartz, A. & Yakir, D. Carbon sequestration in arid-land forest. *Glob. Change Biol.* **9**, 791–799 (2003).
28. Valentini, R. *et al.* Seasonal net carbon dioxide exchange of a beech forest with the atmosphere. *Glob. Change Biol.* **2**, 199–207 (1996).
29. Marcolla, B., Pitacco, A. & Cescatti, A. Canopy architecture and turbulence structure in a coniferous forest. *Bound.-Layer Meteorol.* **108**, 39–59 (2003).
30. Marcolla, B. *et al.* Climatic controls and ecosystem responses drive the inter-annual variability of the net ecosystem exchange of an alpine meadow. *Agric. For. Meteorol.* **151**, 1233–1243 (2011).
31. Spano, D., Snyder, R. L., Sirca, C. & Duce, P. ECOWAT—A model for ecosystem evapotranspiration estimation. *Agric. For. Meteorol.* **149**, 1584–1596 (2009).
32. Montagnani, L. *et al.* A new mass conservation approach to the study of CO2 advection in an alpine forest. *J. Geophys. Res. Atmospheres* **114**, 1–25 (2009).
33. Chiesi, M. *et al.* Modelling carbon budget of Mediterranean forests using ground and remote sensing measurements. *Agric. For. Meteorol.* **135**, 22–34 (2005).
34. Migliavacca, M. *et al.* Using digital repeat photography and eddy covariance data to model grassland phenology and photosynthetic CO2 uptake. *Agric. For. Meteorol.* **151**, 1325–1337 (2011).
35. Dolman, A. J., Moors, E. J. & Elbers, J. A. The carbon uptake of a mid latitude pine forest growing on sandy soil. *Agric. For. Meteorol.* **111**, 157–170 (2002).

36. Kurbatova, J., Li, C., Varlagin, A., Xiao, X. & Vygodskaya, N. Modeling carbon dynamics in two adjacent spruce forests with different soil conditions in Russia. *Biogeosciences* **5**, 969–980 (2008).
37. Sagerfors, J. *et al.* Annual CO2 exchange between a nutrient-poor, minerotrophic, boreal mire and the atmosphere. *J. Geophys. Res. Biogeosciences* **113**, (2008).
38. Ouimette, A. P. *et al.* Carbon fluxes and interannual drivers in a temperate forest ecosystem assessed through comparison of top-down and bottom-up approaches. *Agric. For. Meteorol.* **256–257**, 420–430 (2018).
39. Wofsy, S. C. *et al.* Net Exchange of CO2 in a Mid-Latitude Forest. *Science* **260**, 1314–1317 (1993).
40. Schwarz, P. A. *et al.* Climatic versus biotic constraints on carbon and water fluxes in seasonally drought-affected ponderosa pine ecosystems. *Glob. Biogeochem. Cycles* **18**, (2004).
41. Roman, D. T. *et al.* The role of isohydric and anisohydric species in determining ecosystem-scale response to severe drought. *Oecologia* **179**, 641–654 (2015).
42. Monson, R. K. *et al.* Carbon sequestration in a high-elevation, subalipine forest. *Glob. Change Biol.* **8**, 459â€“478 (2002).
43. Noormets, A. *et al.* Drought during canopy development has lasting effect on annual carbon balance in a deciduous temperate forest. *New Phytol.* **179**, 818–828 (2008).
44. Desai, A. R. Influence and predictive capacity of climate anomalies on daily to decadal extremes in canopy photosynthesis. *Photosynth. Res.* **119**, 31–47 (2014).
45. Anderson-Teixeira, K. J., Delong, J. P., Fox, A. M., Brese, D. A. & Litvak, M. E. Differential responses of production and respiration to temperature and moisture drive the carbon balance across a climatic gradient in New Mexico. *Glob. Change Biol.* **17**, 410–424 (2011).
46. Scott, R. L., Biederman, J. A., Hamerlynck, E. P. & Barron-Gafford, G. A. The carbon balance pivot point of southwestern U.S. semiarid ecosystems: Insights from the 21st century drought. *J. Geophys. Res. Biogeosciences* **120**, 2612–2624 (2015).
47. Baldocchi, D. D., Xu, L. K. & Kiang, N. How plant functional-type, weather, seasonal drought, and soil physical properties alter water and energy fluxes of an oak-grass savanna and an annual grassland. *Agric. For. Meteorol.* **123**, 13–39 (2004).
48. Ueyama, M., Iwata, H. & Harazono, Y. Autumn warming reduces the CO2 sink of a black spruce forest in interior Alaska based on a nine-year eddy covariance measurement. *Glob. Change Biol.* **20**, 1161–1173 (2014).
49. Gough, C. M. *et al.* Sustained carbon uptake and storage following moderate disturbance in a Great Lakes forest. *Ecol. Appl.* **23**, 1202–1215 (2013).
50. Yi, C. *et al.* Observed covariance between ecosystem carbon exchange and atmospheric boundary layer dynamics at a site in northern Wisconsin. *J. Geophys. Res. Atmospheres* **109**, (2004).
51. Tedeschi, V. *et al.* Soil respiration in a Mediterranean oak forest at different developmental stages after coppicing. *Glob. Change Biol.* **12**, 110–121 (2006).
52. Zeller, K. F. & Nikolov, N. T. Quantifying simultaneous fluxes of ozone, carbon dioxide and water vapor above a subalpine forest ecosystem. *Environ. Pollut.* **107**, 1–20 (2000).
53. Dumortier, P. *et al.* Methane balance of an intensively grazed pasture and estimation of the enteric methane emissions from cattle. *Agric. For. Meteorol.* **232**, 527–535 (2017).

54. Humphreys, E. R. *et al.* Carbon dioxide fluxes in coastal Douglas-fir stands at different stages of development after clearcut harvesting. *Agric. For. Meteorol.* **140**, 6–22 (2006).
55. Bergeron, O. *et al.* Comparison of carbon dioxide fluxes over three boreal black spruce forests in Canada. *Glob. Change Biol.* **13**, 89–107 (2007).
56. Coursolle, C. *et al.* Late-summer carbon fluxes from Canadian forests and peatlands along an east-west continental transect. *Can. J. For. Res.* **36**, 783–800 (2006).
57. Hiller, R., Zeeman, M. J. & Eugster, W. Eddy-Covariance Flux Measurements in the Complex Terrain of an Alpine Valley in Switzerland. *Bound.-Layer Meteorol.* **127**, 449–467 (2008).
58. Ammann, C., Spirig, C., Leifeld, J. & Neftel, A. Assessment of the nitrogen and carbon budget of two managed temperate grassland fields. *Agric. Ecosyst. Environ.* **133**, 150–162 (2009).
59. Acosta, M., Darenova, E., Dušek, J. & Pavelka, M. Soil carbon dioxide fluxes in a mixed floodplain forest in the Czech Republic. *Eur. J. Soil Biol.* **82**, 35–42 (2017).
60. McGloin, R. *et al.* Energy balance closure at a variety of ecosystems in Central Europe with contrasting topographies. *Agric. For. Meteorol.* **248**, 418–431 (2018).
61. Wollschläger, U. *et al.* The Bode hydrological observatory: a platform for integrated, interdisciplinary hydro-ecological research within the TERENO Harz/Central German Lowland Observatory. *Environ. Earth Sci.* **76**, 29 (2016).
62. Koebsch, F. *et al.* The impact of occasional drought periods on vegetation spread and greenhouse gas exchange in rewetted fens. *Philos. Trans. R. Soc. B Biol. Sci.* **375**, 20190685 (2020).
63. Vorobevskii, I., Luong, T. T., Kronenberg, R., Grünwald, T. & Bernhofer, C. *Modelling evaporation with local, regional and global BROOK90 frameworks: importance of parameterization and forcing*. https://hess.copernicus.org/preprints/hess-2021-602/ (2021) doi:10.5194/hess-2021-602.
64. Anthoni, P. M. *et al.* Forest and agricultural land-use-dependent CO2 exchange in Thuringia, Germany. *Glob. Change Biol.* **10**, 2005–2019 (2004).
65. Post, H., Hendricks Franssen, H. J., Graf, A., Schmidt, M. & Vereecken, H. Uncertainty analysis of eddy covariance CO_2 flux measurements for different EC tower distances using an extended two-tower approach. *Biogeosciences* **12**, 1205–1221 (2015).
66. Bogena, H. R., Huisman, J. A., Baatz, R., Hendricks Franssen, H.-J. & Vereecken, H. Accuracy of the cosmic-ray soil water content probe in humid forest ecosystems: The worst case scenario. *Water Resour. Res.* **49**, 5778–5791 (2013).
67. Luo, Y. *et al.* Using Near-Infrared-Enabled Digital Repeat Photography to Track Structural and Physiological Phenology in Mediterranean Tree–Grass Ecosystems. *Remote Sens.* **10**, 1293 (2018).
68. El-Madany, T. S. *et al.* Drivers of spatio-temporal variability of carbon dioxide and energy fluxes in a Mediterranean savanna ecosystem. *Agric. For. Meteorol.* **262**, 258–278 (2018).
69. Ilvesniemi, H. *et al.* Long-term measurements of the carbon balance of a boreal Scots pine dominated forest ecosystem. *Boreal Environ. Res.* **14**, 23 (2009).
70. Al-Yaari, A. *et al.* The AQUI Soil Moisture Network for Satellite Microwave Remote Sensing Validation in South-Western France. *Remote Sens.* **10**, 1839 (2018).
71. Granier, A. *et al.* The carbon balance of a young Beech forest. *Funct. Ecol.* **14**, 312–325 (2000).

72. Garbulsky, M. F., Peñuelas, J., Papale, D. & Filella, I. Remote estimation of carbon dioxide uptake by a Mediterranean forest. *Glob. Change Biol.* **14**, 2860–2867 (2008).
73. Tezza, L., Vendrame, N. & Pitacco, A. Disentangling the carbon budget of a vineyard: The role of soil management. *Agric. Ecosyst. Environ.* **272**, 52–62 (2019).
74. Rey, A. *et al.* Annual variation in soil respiration and its components in a coppice oak forest in Central Italy. *Glob. Change Biol.* **8**, 851–866 (2002).
75. Cescatti, A., Marcolla, B., Goded, I. & Gruening, C. Optimal use of buffer volumes for the measurement of atmospheric gas concentration in multi-point systems. *Atmospheric Meas. Tech.* **9**, 4665–4672 (2016).
76. Jacobs, C. M. J. *et al.* Variability of annual CO2 exchange from Dutch grasslands. *Biogeosciences* **4**, 803–816 (2007).
77. Lindroth, A. *et al.* Effects of drought and meteorological forcing on carbon and water fluxes in Nordic forests during the dry summer of 2018. *Philos. Trans. R. Soc. B Biol. Sci.* **375**, 20190516 (2020).
78. Peichl, M., Nilsson, M. B., Pilegaard, K., de Simon, G. & ICOS Ecosystem Thematic Centre. Warm winter 2020 ecosystem eddy covariance flux product from Rosinedal-3. (2021) doi:10.18160/RJ1S-CC2Z.
79. Chi, J. *et al.* The Net Landscape Carbon Balance—Integrating terrestrial and aquatic carbon fluxes in a managed boreal forest landscape in Sweden. *Glob. Change Biol.* **26**, 2353–2367 (2020).
80. Goldstein, A. H. *et al.* Effects of climate variability on the carbon dioxide, water, and sensible heat fluxes above a ponderosa pine plantation in the Sierra Nevada (CA). *Agric. For. Meteorol.* **101**, 113â€“129 (2000).
81. Siqueira, M. B. *et al.* Multiscale model intercomparisons of CO2 and H2O exchange rates in a maturing southeastern US pine forest. *Glob. Change Biol.* **12**, 1189–1207 (2006).
82. Ellsworth, D. S., Oren, R., Huang, C., Phillips, N. & Hendrey, G. R. Leaf and canopy responses to elevated CO2 in a pine forest under free-air CO2 enrichment. *Oecologia* **104**, 139–146 (1995).
83. Amiro, B. D. *et al.* Ecosystem carbon dioxide fluxes after disturbance in forests of North America. *J. Geophys. Res. Biogeosciences* **115**, (2010).
84. Hollinger, D. Y. *et al.* Seasonal patterns and environmental control of carbon dioxide and water vapour exchange in an ecotonal boreal forest. *Glob. Change Biol.* **5**, 891–902 (1999).
85. Miller, R. M., Miller, S. P., Jastrow, J. D. & Rivetta, C. B. Mycorrhizal mediated feedbacks influence net carbon gain and nutrient uptake in Andropogon gerardii. *New Phytol.* **155**, 149–162 (2002).
86. Brunsell, N. A., Nippert, J. B. & Buck, T. L. Impacts of seasonality and surface heterogeneity on water-use efficiency in mesic grasslands. *Ecohydrology* **7**, 1223–1233 (2014).
87. Vickers, D., Thomas, C. & Law, B. E. Random and systematic CO2 flux sampling errors for tower measurements over forests in the convective boundary layer. *Agric. For. Meteorol.* **149**, 73–83 (2009).
88. Nakai, T. *et al.* Characteristics of evapotranspiration from a permafrost black spruce forest in interior Alaska. *Polar Sci.* **7**, 136–148 (2013).

89. Sanchez-Mejia, Z. M. & Papuga, S. A. Observations of a two-layer soil moisture influence on surface energy dynamics and planetary boundary layer characteristics in a semiarid shrubland. *Water Resour. Res.* **50**, 306–317 (2014).
90. Desai, A. R., Bolstad, P. V., Cook, B. D., Davis, K. J. & Carey, E. V. Comparing net ecosystem exchange of carbon dioxide between an old-growth and mature forest in the upper Midwest, USA. *Agric. For. Meteorol.* **128**, 33–55 (2005).
91. Oikawa, P. Y. *et al.* Evaluation of a hierarchy of models reveals importance of substrate limitation for predicting carbon dioxide and methane exchange in restored wetlands. *J. Geophys. Res. Biogeosciences* **122**, 145–167 (2017).
92. Le Quéré, C. *et al.* Global Carbon Budget 2017. *Earth Syst. Sci. Data* **10**, 405–448 (2018).
93. Sun, W. *et al.* Midwest US Croplands Determine Model Divergence in North American Carbon Fluxes. *AGU Adv.* **2**, 1–17 (2021).
94. Haverd, V. *et al. A new version of the CABLE land surface model (Subversion revision r4546), incorporatingland use and land cover change, woody vegetation demography and a novel optimisation-basedapproach to plant coordination of electron transport and carboxylation capacity-limitedphotosynthesis*. https://gmd.copernicus.org/preprints/gmd-2017-265/gmd-2017-265.pdf (2017) doi:10.5194/gmd-2017-265.
95. Melton, J. R. & Arora, V. K. Competition between plant functional types in the Canadian Terrestrial Ecosystem Model (CTEM) v. 2.0. *Geosci. Model Dev.* **9**, 323–361 (2016).
96. Oleson, K. *et al. Technical description of version 4.5 of the Community Land Model (CLM)*. 5912 KB http://opensky.ucar.edu/islandora/object/technotes:515 (2013) doi:10.5065/D6RR1W7M.
97. Tian, H. *et al.* North American terrestrial CO2 uptake largely offset by CH4 and N2O emissions: toward a full accounting of the greenhouse gas budget. *Clim. Change* **129**, 413–426 (2015).
98. Jain, A. K., Meiyappan, P., Song, Y. & House, J. I. CO2 emissions from land-use change affected more by nitrogen cycle, than by the choice of land-cover data. *Glob. Change Biol.* **19**, 2893–2906 (2013).
99. Reick, C. H., Raddatz, T., Brovkin, V. & Gayler, V. Representation of natural and anthropogenic land cover change in MPI-ESM. *J. Adv. Model. Earth Syst.* **5**, 459–482 (2013).
100. Clark, D. B. *et al.* The Joint UK Land Environment Simulator (JULES), model description – Part 2: Carbon fluxes and vegetation dynamics. *Geosci. Model Dev.* **4**, 701–722 (2011).
101. Sitch, S. *et al.* Evaluation of ecosystem dynamics, plant geography and terrestrial carbon cycling in the LPJ dynamic global vegetation model. *Glob. Change Biol.* **9**, 161–185 (2003).
102. Smith, B. *et al.* Implications of incorporating N cycling and N limitations on primary production in an individual-based dynamic vegetation model. *Biogeosciences* **11**, 2027–2054 (2014).
103. Keller, K. M. *et al.* 20th century changes in carbon isotopes and water-use efficiency: tree-ring-based evaluation of the CLM4.5 and LPX-Bern models. *Biogeosciences* **14**, 2641–2673 (2017).
104. Krinner, G. *et al.* A dynamic global vegetation model for studies of the coupled atmosphere-biosphere system. *Glob. Biogeochem. Cycles* **19**, (2005).

105. Guimberteau, M. *et al.* ORCHIDEE-MICT (v8.4.1), a land surface model for the high latitudes: model description and validation. *Geosci. Model Dev.* **11**, 121–163 (2018).
106. Zeng, N., Mariotti, A. & Wetzel, P. Terrestrial mechanisms of interannual CO2 variability. *Glob. Biogeochem. Cycles* **19**, (2005).
107. Kato, E., Kinoshita, T., Ito, A., Kawamiya, M. & Yamagata, Y. Evaluation of spatially explicit emission scenario of land-use change and biomass burning using a process-based biogeochemical model. *J. Land Use Sci.* **8**, 104–122 (2013).
