# Supplementary material for: Increased photosynthesis during spring drought in energy-limited ecosystems
Source: Nat Commun. 2023 Nov 29;14:7828. doi: 10.1038/s41467-023-43430-9 (PMC10687245; doi:10.1038/s41467-023-43430-9)
Supplement: Supplementary file 3 — Reporting Summary [file 41467_2023_43430_MOESM3_ESM.pdf]

nature portfolio

Corresponding author(s): David L. Miller

Last updated by author(s): Nov 3, 2023

Reporting Summary

Nature Portfolio wishes to improve the reproducibility of the work that we publish. This form provides structure for consistency and transparency in reporting. For further information on Nature Portfolio policies, see our Editorial Policies and the Editorial Policy Checklist.

Statistics

For all statistical analyses, confirm that the following items are present in the figure legend, table legend, main text, or Methods section.

n/a	Confirmed	
☐	☒	The exact sample size (n) for each experimental group/condition, given as a discrete number and unit of measurement
☐	☒	A statement on whether measurements were taken from distinct samples or whether the same sample was measured repeatedly
☐	☒	The statistical test(s) used AND whether they are one- or two-sided<br>*Only common tests should be described solely by name; describe more complex techniques in the Methods section.*
☐	☒	A description of all covariates tested
☐	☒	A description of any assumptions or corrections, such as tests of normality and adjustment for multiple comparisons
☐	☒	A full description of the statistical parameters including central tendency (e.g. means) or other basic estimates (e.g. regression coefficient) AND variation (e.g. standard deviation) or associated estimates of uncertainty (e.g. confidence intervals)
☐	☒	For null hypothesis testing, the test statistic (e.g. F, t, r) with confidence intervals, effect sizes, degrees of freedom and P value noted<br>*Give P values as exact values whenever suitable.*
☒	☐	For Bayesian analysis, information on the choice of priors and Markov chain Monte Carlo settings
☒	☐	For hierarchical and complex designs, identification of the appropriate level for tests and full reporting of outcomes
☐	☒	Estimates of effect sizes (e.g. Cohen's d, Pearson's r), indicating how they were calculated

Our web collection on statistics for biologists contains articles on many of the points above.

Software and code

Policy information about availability of computer code

Data collection	All data was compiled from source repositories using open source R packages (see data availability statement).
Data analysis	All data was analyzed in open source R packages and code is available through Github (see code availability statement) and upon reasonable request. Software include: R 4.1.3 (with packages tidyverse 1.3.1, ggpmisc 0.4.5, ggpubr 0.4.0, Kendall 2.2, mapproj 1.2.8, maps 3.4.0, ncdf4 1.19, raster 3.5-15, RColorBrewer 1.1-3, reshape2 1.4.4, rnaturalearth 0.1.0, rnaturalearthdata 0.1.0, sf 1.0-7, shadowtext 0.1.2, sp 1.4-6, R.matlab 3.6.2, trend 1.1.4, zyp 0.10-1.1), RStudio 2022.07.1, QGIS 3.22, Matlab R2022a, Adobe Illustrator 2022

For manuscripts utilizing custom algorithms or software that are central to the research but not yet described in published literature, software must be made available to editors and reviewers. We strongly encourage code deposition in a community repository (e.g. GitHub). See the Nature Portfolio guidelines for submitting code & software for further information.

Data

Policy information about availability of data

All manuscripts must include a data availability statement. This statement should provide the following information, where applicable:
- Accession codes, unique identifiers, or web links for publicly available datasets
- A description of any restrictions on data availability
- For clinical datasets or third party data, please ensure that the statement adheres to our policy

Eddy covariance data is available from:

FLUXNET2015: https://fluxnet.org/data/fluxnet2015-dataset/
ICOS Drought-2018: https://doi.org/10.18160/YVR0-4898
ICOS Warm Winter 2020: https://doi.org/10.18160/2G60-ZHAK
ONEFlux Beta: https://ameriflux.lbl.gov/data/download-data-oneflux-beta/
Other data sources are available from:
CRU NCEP: https://rda.ucar.edu/datasets/ds314.3/
CRU TS v 4.05: https://crudata.uea.ac.uk/cru/data/hrg/
FLUXCOM: https://www.fluxcom.org/CF-Download/
GOSIF GPP: https://globalecology.unh.edu/data/GOSIF-GPP.html
MODIS GPP (through NASA AppEEARS): https://appeears.earthdatacloud.nasa.gov/
TerraClimate: https://www.climatologylab.org/terraclimate.html
TRENDY: https://blogs.exeter.ac.uk/trendy/

Research involving human participants, their data, or biological material

Policy information about studies with human participants or human data. See also policy information about sex, gender (identity/presentation), and sexual orientation and race, ethnicity and racism.

Reporting on sex and gender	Not applicable
Reporting on race, ethnicity, or other socially relevant groupings	Not applicable
Population characteristics	Not applicable
Recruitment	Not applicable
Ethics oversight	Not applicable

Note that full information on the approval of the study protocol must also be provided in the manuscript.

Field-specific reporting

Please select the one below that is the best fit for your research. If you are not sure, read the appropriate sections before making your selection.

☐ Life sciences ☐ Behavioural & social sciences ☒ Ecological, evolutionary & environmental sciences

For a reference copy of the document with all sections, see nature.com/documents/nr-reporting-summary-flat.pdf

Ecological, evolutionary & environmental sciences study design

All studies must disclose on these points even when the disclosure is negative.

Study description	Our study addresses how energy-limited ecosystems can increase productivity during spring drought conditions, and we make comparisons across eddy covariance, terrestrial biosphere models, and remote sensing data products.
Research sample	We used 61 sites in spring, 62 in summer, and 63 in fall for our main collection of minimum 10 years of data; for the minimum of 5 years of data, we had 112 sites (see Methods). There are 5 remote sensing productivity products (MODIS Terra, MODIS Aqua, GOSIF-GPP, FLUXCOM-RS, and FLUXCOM-RS+METEO) and 14 terrestrial biosphere models from TRENDY v6. We used TerraClimate for gridded climate information for information with CRU v 4.05 for supplemental aridity analysis (to test different spatial scale for models). Please see Supplemental tables for complete lists and information for sites and models.
Sampling strategy	Data from eddy covariance sites were sampled for sites that had at minimum 5 years of data, with a minimum of 10 years of data for the final list of high quality sites. This site selection was used to extract individual sites from gridded products, and using gridded products for land area for north of 30 N, filtered based on MODIS land cover labels for regions of vegetation.
Data collection	Eddy covariance was collected based on the community standards in FLUXNET. Other gridded products were collected from publicly available repositories (see data availability statement). Data collection at individual eddy covariance sites was performed by site PIs, and relevant references are listed in Supplemental Table S1.
Timing and spatial scale	The spatial extent is a combination of individual field sites and gridded products that are north of 30 N. Eddy covariance data is from 1992 to 2020, depending on the site and the source dataset (e.g., FLUXNET2015). The terrestrial biosphere model outputs were extracted for the time range of 1992 to 2016. The remote sensing dataset are of different time ranges depending on the product, from 1992 to present for FLUXCOM RS+meteo, and other products from approximately the year 2000 (more specific details are in the Methods section). Gridded climate data, including TerraClimate and CRU, were used to calculate aridity (wetness index) for the time range of 1981 to 2010.

Data exclusions	Eddy covariance data was screened based on quality control flags based on community standards in FLUXNET, and other known events (e.g., fires; see Methods for more details).
Reproducibility	The study is a data analysis and can be reproduced based on code availability.
Randomization	Randomization was not relevant to the study because we used all available data and did not randomize or sub-select within the available data.
Blinding	Blinding was not relevant to the study because we did not conceal groups of individuals or data sets from other groups or data sets.

Did the study involve field work? ☐ Yes ☒ No

Reporting for specific materials, systems and methods

We require information from authors about some types of materials, experimental systems and methods used in many studies. Here, indicate whether each material, system or method listed is relevant to your study. If you are not sure if a list item applies to your research, read the appropriate section before selecting a response.

Materials & experimental systems

n/a	Involved in the study
☒	☐ Antibodies
☒	☐ Eukaryotic cell lines
☒	☐ Palaeontology and archaeology
☒	☐ Animals and other organisms
☒	☐ Clinical data
☒	☐ Dual use research of concern
☒	☐ Plants

Methods

n/a	Involved in the study
☒	☐ ChIP-seq
☒	☐ Flow cytometry
☒	☐ MRI-based neuroimaging
